# Batch Effect Confounding Leads to Strong Bias in Performance Estimates Obtained by Cross-Validation

**DOI:** 10.1371/journal.pone.0100335

**Published:** 2014-06-26

**Authors:** Charlotte Soneson, Sarah Gerster, Mauro Delorenzi

**Affiliations:** 1 SIB Swiss Institute of Bioinformatics, Lausanne, Switzerland; 2 Ludwig Center for Cancer Research, University of Lausanne, Lausanne, Switzerland; 3 Oncology Department, University of Lausanne, Lausanne, Switzerland; Queen's University Belfast, United Kingdom

## Abstract

**Background:**

With the large amount of biological data that is currently publicly available, many investigators combine multiple data sets to increase the sample size and potentially also the power of their analyses. However, technical differences (“batch effects”) as well as differences in sample composition between the data sets may significantly affect the ability to draw generalizable conclusions from such studies.

**Focus:**

The current study focuses on the construction of classifiers, and the use of cross-validation to estimate their performance. In particular, we investigate the impact of batch effects and differences in sample composition between batches on the accuracy of the classification performance estimate obtained via cross-validation. The focus on estimation bias is a main difference compared to previous studies, which have mostly focused on the predictive performance and how it relates to the presence of batch effects.

**Data:**

We work on simulated data sets. To have realistic intensity distributions, we use real gene expression data as the basis for our simulation. Random samples from this expression matrix are selected and assigned to group 1 (e.g., ‘control’) or group 2 (e.g., ‘treated’). We introduce batch effects and select some features to be differentially expressed between the two groups. We consider several scenarios for our study, most importantly different levels of confounding between groups and batch effects.

**Methods:**

We focus on well-known classifiers: logistic regression, Support Vector Machines (SVM), k-nearest neighbors (kNN) and Random Forests (RF). Feature selection is performed with the Wilcoxon test or the lasso. Parameter tuning and feature selection, as well as the estimation of the prediction performance of each classifier, is performed within a nested cross-validation scheme. The estimated classification performance is then compared to what is obtained when applying the classifier to independent data.

## Introduction

Every day, large quantities of data are generated by biological and medical labs all over the world. Largely facilitated by online repositories such as Gene Expression Omnibus (http://www.ncbi.nlm.nih.gov/geo/ [1]) and ArrayExpress (http://www.ebi.ac.uk/arrayexpress/ [2]), many of these data sets are made freely available for other researchers to use. This has inspired many investigators to design studies based entirely on public data or to use public data in combination with their own data, to increase the sample size and thereby hopefully the power to draw conclusions (e.g., [3]). One field where the data sharing practices are well developed and standardized is the one of high-throughput profiling of gene expression data, which is the main motivation behind the current study. However, the line of reasoning pursued in this paper, as well as the main conclusions, are likely valid for many different (biological and non-biological) types of data.

Most of the publicly available gene expression data have been generated using *expression microarrays* measuring the expression of thousands of genes in a single assay (see e.g. [4]), although next-generation sequencing-based profiling of gene expression (RNA-seq) is becoming increasingly common. The processing of microarray data is well established and standard analysis pipelines are available, but still there are (well-known) pitfalls. For example, the measured gene expression levels are very sensitive to external factors such as the technician running the experiment, the reagent batch, or the time of the day when an assay was processed [5]. Such systematic errors, related to technical aspects, are often referred to as *batch effects* (see [6] for a comprehensive discussion). In this article, we use batch effects in a wide sense, to represent any type of systematic bias between groups treated at different timepoints or under different external conditions. In other words, the bias introduced by a batch effect “may be defined as unintentional, systematic erroneous association of some characteristic with a group in a way that distorts a comparison with another group” [5]. These biases can appear between data sets from completely different studies, but also within single studies, where time and capacity restrictions may imply that it is not always possible to process all samples under identical conditions. Typically, batch effects affect different variables in different ways, and are usually not eliminated by common between-sample normalization methods [7], [8]. Microarray experiments (just like any other experiment) need to be carefully planned in order to avoid confounding among potentially influential variables. If, for example, all stage III patients in a cancer study are female and all stage II patients are male, we will not be able to distinguish the gender effect from the stage effect on our results. In such situations, we say that the gender variable is *confounded* with the stage variable [7]. The confounding can be strong, as in the mentioned case, or weaker, e.g. if the stage II population is enriched with male subjects, but the two categories are not completely overlapping. Complete absence of confounding would mean that the men (and women) are equally distributed in the stage II and stage III populations. Within a single study, confounding between the effect of the main outcome variable of interest and the effect of other influential variables can often be minimized with careful experimental design (see e.g. [9]). However, even in well-designed studies, unexpected events like dropouts or technical sample preparation differences can disrupt the original design and introduce confounding. When combining different data sets generated by different groups or at different times, it is even more difficult since no single person has control over the entire design. The increased sharing of data between researchers through public data repositories further implies that it is of utmost importance for every research group to document potential confounding variables, to allow other researchers to estimate the degree of confounding and design their experiment appropriately.

Systematic differences between the combined data sets can have a big influence on the subsequent analysis and can, if not properly dealt with, lead to spurious findings as well as conceal true effects [6], [7], [10], [11]. The current study focuses on the impact of batch effects on the ability to build and evaluate the performance of a classifier based on gene expression data. Construction of classifiers, with the aim to assign samples to groups or predict some other trait of interest, is one of the most common goal in gene expression studies. Many studies have focused on different methodologies and pipelines for training a classifier, with the conclusion that there is no such thing as *the one and only best/correct classification procedure* [12], [13]. Yet other researchers dealt with the concept of variable selection and proper assessment of the performance of classifiers [12], [14]–[20].

Due to the high prevalence and potentially strong impact of batch effects, several authors have proposed methods attempting to eliminate their effects on observed data [21]–[24]. Studies comparing several of these tools have led to the conclusion that the performance of most approaches is similar [8], [25]. The majority of these methods assume that the confounding factor(s) are known to the investigator. Sometimes, however, despite careful experimental design there may be unrecorded or poorly documented variables that are confounded with the outcome of interest. Recently, several methods have been presented for estimating and eliminating such unknown batch effects, mainly in the context of differential expression analysis [26], [27]. We expect batch effect removal methods to be most effective when the degree of confounding between the batch variable and the endpoint of interest is low, so that the gene expression effects attributable to the two variables can be disentangled.

In this study, we investigate what results to expect when a classifier is built and evaluated on a data set that may contain batch effects, potentially (partly or entirely) confounded with the class variable. As an example, consider a situation where we are interested in building a classifier to distinguish cancer patients (group 1) from healthy volunteers (group 2), and we combine one public data set consisting of only healthy volunteers with our own data set consisting of only patients. In such a situation, the fact that the patients and the healthy volunteers come from different data sets may introduce apparent differences between them that are not truly related to the disease, and that thus may fail to generalize or be replicated in other studies. The main goal of this study is to evaluate whether the presence of a confounding factor introduces a bias in “internal” classifier performance estimates obtained via cross-validation compared to the actual performance of the classifier on external data. Subsequently, we are also interested in whether, in the presence of a confounding factor, a commonly used batch effect removal method is able to eliminate the potential bias it introduces. The importance of the study follows from the observation that internal cross-validation estimates are often used in practice as proxys for the true (external) performance (that we expect if the classifier is applied to an independent data set), since researchers typically want to use as many samples as possible to construct the classifier. Simulation studies like this one are therefore important to estimate the accuracy of the internal measure. We demonstrate that running a standard pipeline of statistical tools in cases where there is strong inherent bias in the input data can give very misleading classification performance estimates, and that not all experimental design problems can be corrected retrospectively. This stresses the need for careful planning before an experiment is performed, in order to avoid batch effect confounding with the endpoint of interest as much as possible.

Our input data is simulated, which means that we have access to the ground truth to estimate the classifiers' performance. We restrict ourselves to situations with two groups (binary classification). In this setting, we perform all steps from the simulated data (normalized and 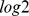 -transformed) to the final classifier – including batch effect removal, gene selection, training the classifier and evaluating the classifier's performance – according to the state of the art in the field. Our study covers two different approaches for gene selection and four different classifiers. The performance of each classifier is evaluated on the training data by the means of nested cross-validation [12], [18]–[20] as well as on external data. We use ComBat [23] as batch effect removal method. Although the setting may seem specific to ComBat and cross-validation, the addressed issue is more general. We expect that the major conclusions of this study also apply to other similar batch effect correction models and different sampling approaches to estimate the performance of a classifier.

We find that in data sets where there are no genes that are truly differentially expressed between the two groups, the internal cross-validation performance estimate is only approximately unbiased when the batch effect is completely non-confounded with the class labels. Eliminating the batch effects can not correct the bias found in other settings. For data sets where some genes are truly differentially expressed, we can use the cross-validation performance estimate as a surrogate for the true performance as long as the level of confounding is not too large. Eliminating the batch effect results in improved classification performance for low levels of confounding.

## Materials

**Figure 1:**
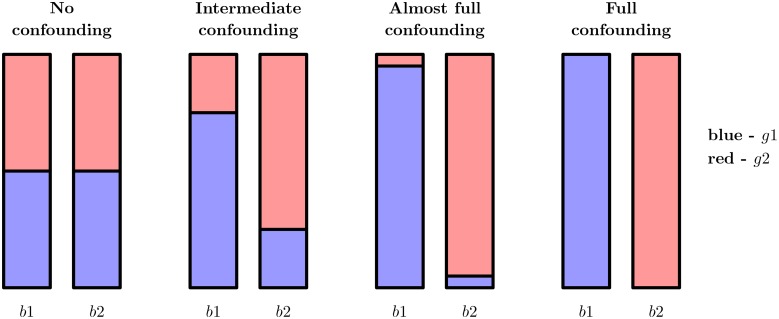
The four confounding levels considered in this study. The two bars for each confounding level correspond to the two batches. The different colors correspond to the two experimental groups (e.g., control and treated). The height of the respective bars illustrate the fraction of the samples belonging to each category. In addition to the four situations shown in this figure, we also consider data sets without batch effect at all, that is, where all samples are generated from the same batch.

The simulated data is generated based on a (background corrected, normalized and log2 transformed) gene expression matrix (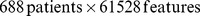, E-MTAB-990) from the PETACC-3 clinical trial [28]. From this we randomly drew 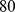 and 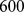 samples to generate training and validation data sets, respectively. The samples were split into two groups (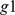 and 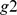). This split was balanced (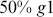 and 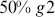). For the training data set, we simulated processing in two batches (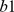 and 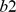), with varying degree of confounding between the batch assignment and the outcome of interest (the sample group). We distinguished four levels of confounding: none (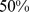 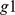 samples in 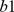, 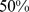 in 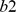; same for 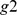 samples), intermediate (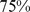 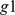 samples in 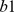, 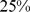 in 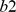; vice versa for 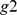 samples), strong (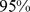 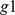 samples in 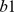, 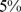 in 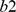; vice versa for 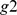 samples) and full (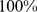 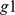 samples in 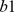, 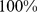 of 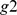 samples in 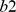). Figure 1 illustrates the four confounding levels.

We simulated *null* data sets where none of the features were differentially expressed between the two groups, as well as data sets where approximately 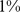 of the features underwent a change in expression between the two sample groups (the *alternative* setting). Furthermore, we simulate both training data sets where the two batches behave the same (that is, where there is no batch effect) and data sets where the two batches behave differently (that is, where there is a batch effect). For training data sets with batch effect, we simulated 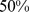 of the features to be affected by it. This is in line with the results of [7], who found between 32.1% and 99.5% of the variables in the data sets they studied to be associated with the batch effect. For each simulation setting, we simulated 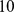 replicate data sets. All in all we trained the classifiers on data from 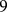 different settings (with 10 replicates for each setting).

All generated *validation* data sets are balanced and are not affected by batch effects, which is motivated by a desire to generate validation data sets that are representative of a general population. The influence of batch effects between the training and validation sets was examined by [8] and [11], who showed that cross-batch prediction works relatively well when the outcome variable is not heavily confounded with the batch variable (that is, if the training data set and the external validation data set have similar sample composition). We generated validation data sets both with and without truly differentially expressed genes. The parameter settings as well as a detailed description of the simulation procedure are provided in Supporting Information S1. In addition, the R code [29] used to simulate the data is also provided in Supporting Information S2.

## Methods

### Data preprocessing

To eliminate the effect of the confounding factor on the training data set, we use ComBat [23], which employs an empirical Bayes approach to estimate a location and scale parameter for each gene in each batch separately, and adjusts the observed expression values based on the estimated parameters. The use of both location and scale adjustment means that both additive and multiplicative batch effects can be eliminated, and the built in empirical Bayes step improves the performance for small sample sizes by pooling information across the genes. Previous studies have shown that ComBat is able to eliminate batch effects in several different situations, and performs at least as well as other batch effect removal methods [25]. We apply ComBat with the confounding factor as the ‘batch’ variable and the known group labels as a covariate. In case the batch factor is completely confounded with the group labels, we eliminate the batch effect without including any covariates in the model. Batch effect removal is often used as a first step, to harmonize different data sets before building a classifier. Hence, in this study, the batch effect removal is always applied to the entire data set (that is, before splitting the data in the cross-validation step).

### Cross-validation

The main theme in this paper is the construction of *classifiers*. A classifier consists of a collection of *predictor variables* and a *classification rule* such that, given observed values of the predictor variables for a sample, we can plug them into the classification rule and based on the result assign a *group label* to the sample. To build such a classifier in a supervised setting, we need a *training data set*, consisting of samples for which we are given the observed values of a number of variables as well as the true group labels. This data is used to select appropriate predictor variables from the variable pool and to construct the classification rule. The goal, however, is to obtain a classifier that will work well for predicting the group labels of *new* samples, that are not part of the training set. In fact, we can even claim that predicting the group labels of the samples in the training set correctly is meaningless since the true group labels are already known. To measure the performance of a classifier constructed from a training set, we thus need to apply it to an independent *test set* that is representative of the data sets for which we want to apply our classifier, but for which the true group labels are known. In this way, we can record the agreement between the labels predicted by the classifier and the true labels. This will tell us how well the classifier can be expected to work in general on independent test data sets (where we do not know the true group labels).

In practical situations, we typically want to use all the available data to construct the classifier. Various methods have been proposed to artificially generate training and test sets from a single data set. Cross-validation is one of the most widely used such approaches, and involves randomly splitting the data set into 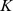 parts (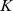 is called the *fold*), and using one of the parts as the test set and the remaining 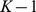 parts as the training set. This procedure is repeated until all 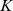 parts have been used once as a test set. Other methods, such as the bootstrap, are also frequently used to generate artificial training and test data sets. Moreover, some classification methods (like the random forest) include a resampling step as part of the model building, which allows the researcher to obtain an “out-of-bag” estimate of the predictive ability already from the model building phase.

Cross-validation is used for many different purposes, arguably the most common ones being to determine the optimal value of hyperparameters for a classifier and to estimate the performance we can expect from a given classification procedure if applied to independent data.

#### Biases of cross-validation estimates

Since the estimates obtained through cross-validation are derived from subsets of the original data set, with different numbers of samples than in the whole set, there will be an inherent bias in these estimates that depends on the value of 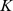. Estimates obtained through leave-one-out cross-validation (LOOCV, or 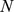 -fold cross-validation where 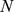 denotes the number of samples) are less biased, but in contrast have higher variance than estimates obtained with smaller folds [30].

We use *stratified* cross-validation, meaning that the fraction of samples from each class is kept as constant as possible across the different cross-validation folds. This ensures for example that each class is present in all training sets.

In this study we focus on the additional biases that may result if the data set used to build the classifier (and thus used as the basis for the cross-validation) is not representative of the collection of new data sets to which the classifier will eventually be applied. In this case, as we will see, the performance estimate obtained from the cross-validation can be far from the actual performance of the classifier on a new independent data set.

#### Cross-validation scheme

**Figure 2:**
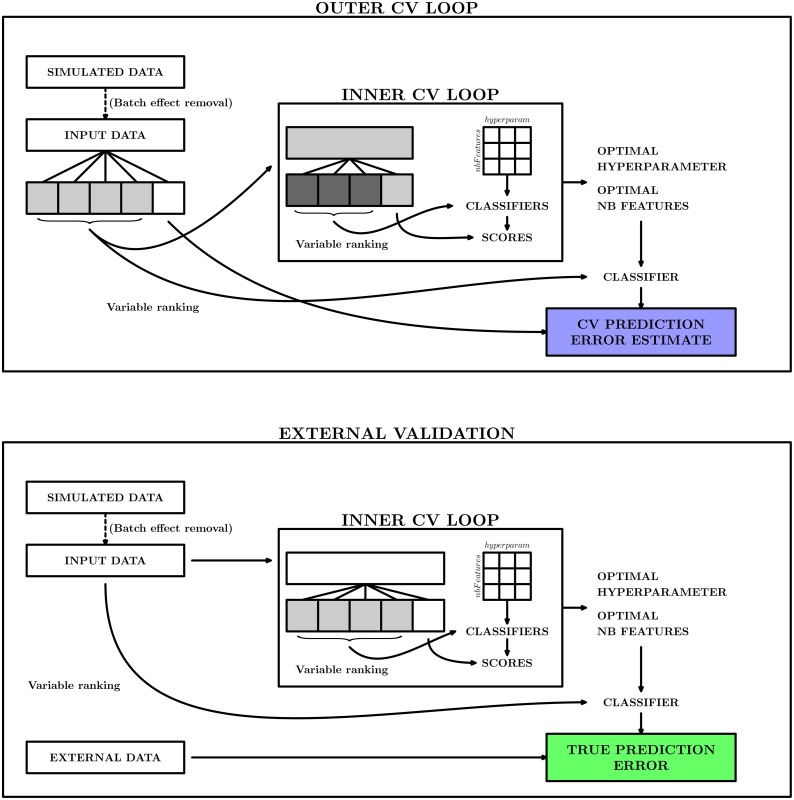
The cross-validation scheme employed in the study. The upper panel illustrates the combination of the inner cross-validation loop, which is used to estimate the optimal combination of the classifier hyperparameter and number of features, and the outer cross-validation loop, which is used to estimate the predictive performance of the constructed classifier. The lower panel shows how the final classifier is built on the whole input data set, and its performance is estimated on an external validation data set. The bias of the estimate from the cross-validation procedure is obtained by comparing the values in the two colored boxes.

In the present study, we apply the cross-validation procedure in a nested, or two-level, fashion as illustrated in the top panel of Figure 2. The purpose of the *outer* cross-validation loop is to provide an estimate of the classification performance of a constructed classifier. The *inner* cross-validation loop, in contrast, is used to build the classifier, that is, to select the optimal combination of hyperparameters and the subset of predictor variables for the classifier. To distinguish the training and test sets from the two levels, we denote the data sets generated in the outer cross-validation loop the *outer* training and test sets, respectively. Given that the fold of the outer cross-validation is 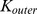, and that the number of samples in the original data set is 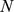, each outer training set will consist of approximately 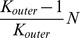 samples, and the corresponding outer test set consists of the remaining 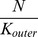 samples. Similarly, the training and test sets generated in the inner cross-validation loop are denoted *inner* training and test sets. Note that since the cross-validation procedure is nested, each pair of inner training and test sets will be generated from, and thus be subsets of, one of the outer training sets. The fold of the inner cross-validation is denoted 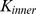.

In each round of the outer cross-validation loop, we thus create an outer training set and an outer test set. On the outer training set, we then apply the inner cross-validation procedure. More precisely, for each inner training set we build several classifiers (with different numbers of predictor variables and/or hyperparameter values). Each of the classifiers is applied to the corresponding inner test set, and the classification performance is recorded. This is repeated for all inner training sets, and the observed performances are averaged across the inner test sets. The combination of hyperparameter value and number of predictor variables giving the best averaged predictive performance is returned from the inner cross-validation loop. Bearing in mind that the combination was chosen since it performed best in the inner cross-validation loop, the performance estimate obtained from the inner cross-validation is biased. It is an overoptimistic estimate of the actual performance of the classifier. Thus, to obtain a better estimate of the performance, a new classifier is built on the outer training set using the selected optimal hyperparameter value and number of variables. This new classifier is then applied to the corresponding outer test set. The predictive performance obtained for this test set is recorded. The final cross-validation estimate of the classification performance is obtained by averaging the estimates obtained for each of the 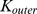 outer test data sets.

**Figure 3:**
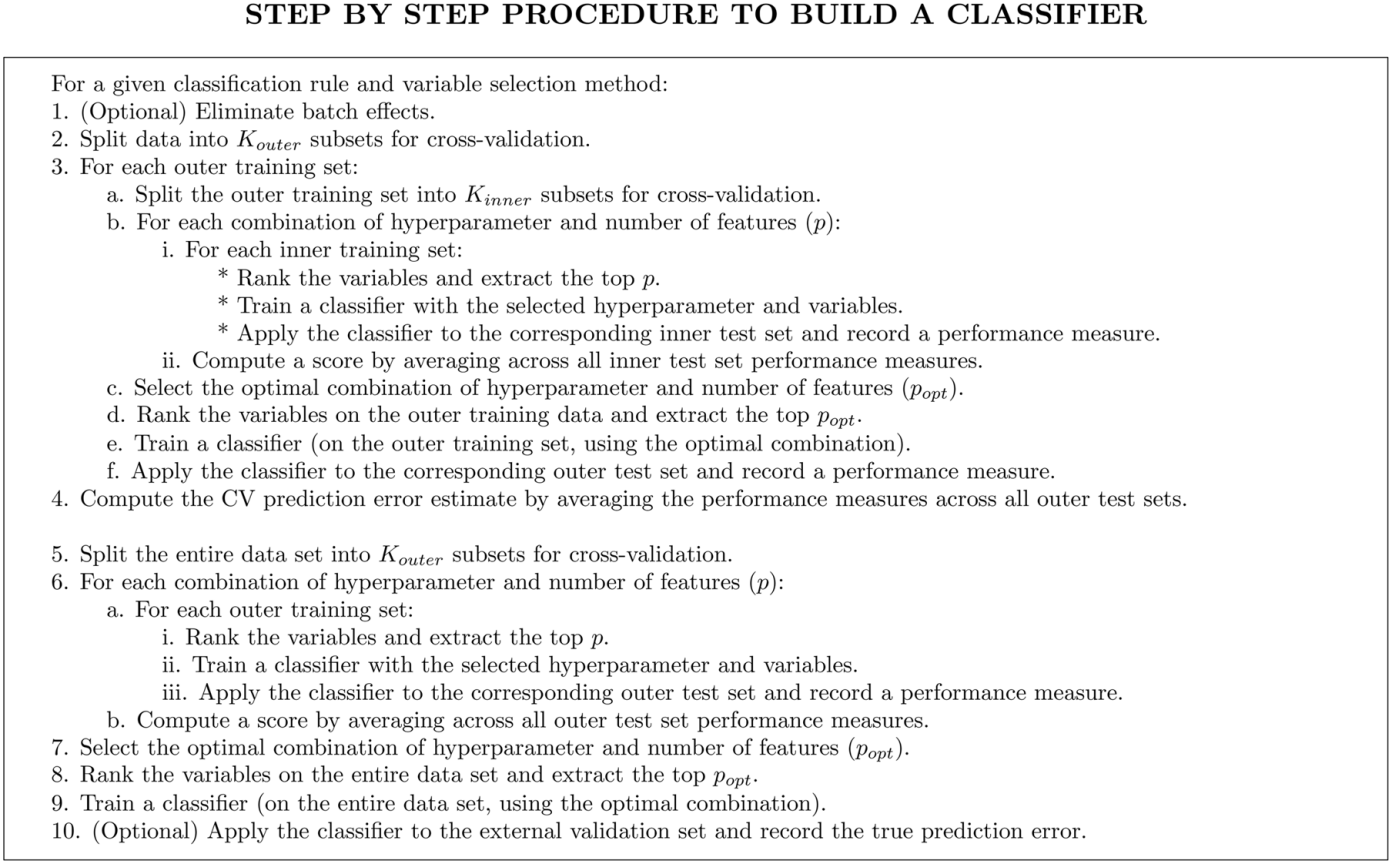
Step-by-step description of the flowchart illustrated in Figure 2. The code used to produce the results presented in this manuscript is provided in Supporting Information S2.

It is important to note that this value is *not* an estimate of the performance of a *specific* classifier (i.e., with a specific set of predictors and a specific classification rule), since different classifiers are built in each round of the outer cross-validation loop. Rather, the value obtained from the cross-validation provides an estimate of the performance of a classifier generated through a specified *workflow* (defined by the hyperparameter and predictor selection procedure defined by the inner cross-validation loop). We compare the performance estimate obtained by the outer cross-validation procedure (perf_*CV*_) to what we consider the “true” performance (perf_*true*_). We obtain perf_*true*_ by building a classifier on the whole original training data set (following the same procedure as above), and applying it to an independent validation data set. This workflow is depicted in the lower panel of Figure 2. If perf_*CV*_ and perf_*true*_ are similar, we conclude that the cross-validation based estimate is unbiased, and that perf_*CV*_ provides a useful estimate of the real performance of our classifier. Conversely, if the two values are far from each other, the cross-validation estimate does not say much about the actual performance of the classifier, and consequently can be quite misleading in practical situations. The whole procedure used to build and evaluate a classifier is outlined in Figure 3.

### Measuring the performance of a classifier

Many measures have been proposed for quantifying the performance of a classifier. The simplest measure is the *misclassification rate*, which is defined as the fraction of the samples that are assigned the wrong label by the classifier. This often works well but can be misleading when the groups are of very different size.

Since all our examples are on balanced data, we present the results in the form of the misclassification rate. Results for other performance measures can be found in Supporting Information S1.

### Classification rules

As noted above, a classifier consists of a collection of predictor variables and a classification rule to combine the observed predictor values and yield a predicted group label. In this section we briefly describe the four approaches we use to build prediction rules, and the next section outlines the methods for selecting predictor variables. We consider four classification approaches in this study; random forests, support vector machines, k-nearest neighbor classifiers and penalized logistic regression.

The random forest classifier was proposed by Breiman [31]. The algorithm uses subsampling of the samples of a data set to generate a large number of similar data sets, and builds a classification tree for each of them. A new sample is then passed through all the classification trees, and voting among their predictions determines its final group label. We built the random forest classifiers using the rfCMA function in the CMA package [32] for R [29]. This implicitly calls functions from the randomForest R package [33]. With default parameters, which were used in this study, the random forest consists of 500 trees. In each branching point, a subset of the available variables are considered as potential split variables. The size of the subset is equal to the square root of the number of variables.

Support vector machines (SVMs) [34] attempt to construct a linear hyperplane that separates the samples into different groups. In doing so, the distance from the hyperplane to the closest point (the margin) is maximized. In most practical applications perfect separation is not feasible, and the objective function is defined as a tradeoff between maximizing the margin and minimizing the distance between the hyperplane and any misclassified points. The tradeoff between the two terms is governed by a cost parameter (usually denoted 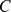). In our experiments, we built SVMs with 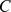 taking values from 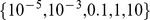. The optimal value of this hyperparameter is selected in the inner cross-validation loop (see Figure 2). We built SVMs using the svmCMA function in the CMA R package. This implicitly calls functions from the e1071 R package [35].

The k-nearest neighbor (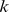 NN) algorithm classifies a new sample by finding the 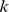 closest samples from the training set, and uses voting among them to assign a group label to the new sample. The performance depends on the hyperparameter 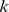 (the number of neighbors), which we select in the inner cross-validation loop. We allow 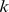 to take values from 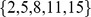. We built the 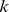 NN classifiers using the knnCMA function in the CMA R package. This implicitly calls functions from the class R package [36].

Given a two-class problem, logistic regression models the natural logarithm of the odds of belonging to class 1 as a linear combination of the predictor variables. As regression models in general, logistic regression is sensitive to collinearity among predictors, and thus a ridge penalization parameter 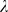 is imposed. Using the inner cross-validation procedure, we select the optimal value of 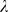 from 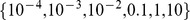. We fit the logistic regression models using the plrCMA function in the CMA R package.

Some classifiers may work better if all predictor variables are on similar scales [37]. For this reason, we z-transform all variable values (that is, we subtract the mean value and divide by the standard deviation) before building a classifier. The scaling parameters (mean and standard deviation for each variable) are always derived from the (outer or inner, respectively) *training* set, and the corresponding *test* set is scaled using the same parameters. This is important to account for potential differences in the class composition of the training and test sets. Results with other normalization procedures are very similar to the ones obtained with the z-transform.

### Selection of predictor variables

As described above, in the inner cross-validation loop, we compare classifiers built on different collections of variables. The set of predictor variables used in a classifier is determined in one of two ways. In the first approach, we use the 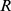 top-ranked variables from a Wilcoxon test comparing the two classes, where 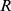 is chosen from 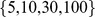. In the second approach, we apply lasso regression [38] with the regularization parameter (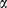) selected from 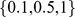, and retain all variables with non-zero regression coefficient. The variable selection is performed by the function GeneSelection from the CMA R package. Note that the variable selection is performed before the classification rule is constructed, and hence all classifiers are built on the same set of variables. However, not all variables may be explicitly used in the classification rules.

It is important that the variable selection is performed only on the training data that will be used to build the classifier. The test set that will be used to evaluate the classifier is not allowed to influence the variable selection, since this could potentially introduce a bias in the performance estimation [20]. Hence, we apply the variable selection to each training set independently, before building a classifier using any of the methods discussed in the previous paragraph.

## Results

### Simulation study, null case

Under the *null* simulation setting – when there are no variables that are truly differentially expressed between the two classes we want to discriminate – it is reasonable to assume that any classifier built on the data will be no better than random guessing when it comes to assigning new samples to the correct group. However, let us assume that there are other factors (we will refer to all such effects as batch effects) that are confounded with the group label in the training data and that affect the values of the variables. For example, imagine a situation where most of the patients treated with drug A (i.e., sample group 1) come from one data set, and most of the samples treated with drug B (sample group 2) come from another data set. In another situation, the patients treated with drug A could be significantly older than those treated with drug B. Confounding factors like these may affect certain genes in such a way that we observe a difference between the two sample groups in the training data, which are not truly related to the factor we are interested in (above, the difference between the two drugs). This means that it may very well be possible to build a classifier that works well on this specific data set, but since these variables are not truly linked to the differences between the drugs, but rather to technical effects, they are unlikely to hold up as good discriminators in another data set. Moreover, the estimate of the predictive performance obtained through the cross-validation procedure on the training set may be far from accurate. Note that in this context, we consider also batch effects that are not necessarily known to the investigator.

**Figure 4:**
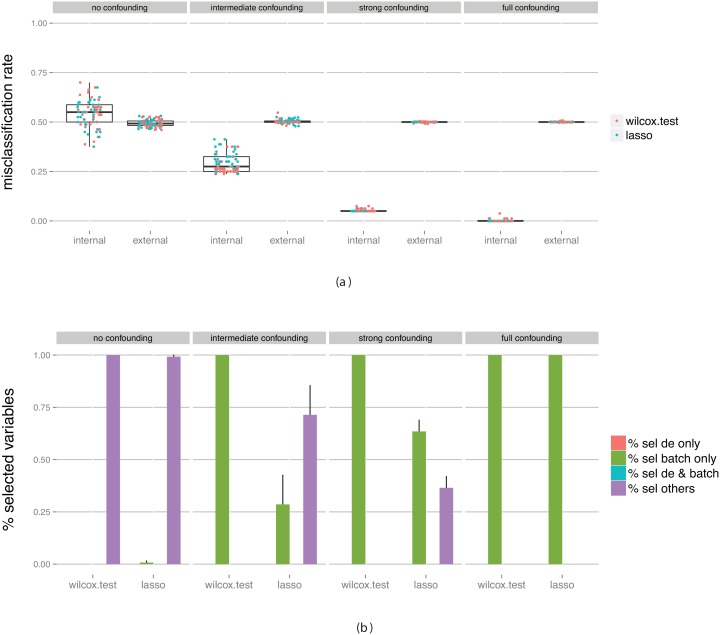
Evaluation of classifiers built on data without truly differentially expressed genes between the classes, but with a batch effect with various degree of confounding with the class labels. (a) Estimated predictive performance from the outer cross-validation (internal) and obtained by applying the constructed classifier to an external test set (external). (b) The fraction of predictor variables selected for the final classifier that were simulated to be differentially expressed and/or associated with the batch. The bars summarize results across all classifiers and all data set replicates. The bar heights represent the average fraction of variables extracted from each category, and the error bars extend one standard deviation above the average. Note that since there are no truly differentially expressed genes in this data set the height of the two corresponding bars is zero.

Figure 4(a) shows the estimated misclassification rate obtained from the cross-validation on the training data set (the *internal* measure) as well as the “true” performance obtained by applying the final classifier to an independent data set (the *external* measure). We are interested in knowing whether the internal measure is an accurate reflection of the external measure. In the figures, we have combined the results for all four evaluated classification algorithms (SVM, RF, logistic regression and kNN), since they behave similarly. In Supporting Information S1 we show figures where the dots are colored by classification algorithm rather than by variable selection algorithm.

The training set is simulated to contain a batch effect, which is not present in the validation data. Moreover, we assume that we are unaware of this batch effect, and thus do not make any attempt to eliminate it at this stage. The four columns of Figure 4(a) correspond to varying levels of confounding between the class labels and the batch factor. In the leftmost panel, the batch factor is not confounded with the class labels, which means that the class labels are equally distributed between the two batches (see Figure 1). In practice, this would correspond e.g. to a situation where we combine two data sets, each containing equal fractions of patients treated with drug A, and equal fractions of patients treated with drug B. In the rightmost panel, there is full confounding between the batch factor and the group labels. In other words, it would correspond to a situation where all patients treated with drug A come from one dataset and all patients treated with drug B come from another dataset. The panels in between correspond to intermediate levels of confounding.

As expected, in all cases, the performance of the classifiers when applied to the external validation set is not better than chance, with an average misclassification rate close to 50%. However, the cross-validation estimate (the ‘internal’ measure) depends strongly on the level of confounding between the batch and the group labels. When there is no confounding (that is, if the samples from each group are evenly distributed between the two batches), the cross-validation estimate is almost unbiased, although the variance is larger than for the external measure. In other words, the performance estimate provided by the cross-validation is a useful proxy for the true performance. As the degree of confounding increases (moving towards the right in the figure), the cross-validation performance estimate becomes increasingly over-optimistic, and with full confounding the cross-validation procedure estimates the misclassification rate to 0 (that is, all samples can be correctly classified). This is not surprising since in the case of full confounding, there is no way to discriminate the batch effect from a true group difference in the training data set. The results observed in the presence of confounding thus imply that the performance estimate obtained by the cross-validation is in fact far from the performance we can expect if we apply the classifier to an external data set, and thus rather misleading.

The type of variables selected for the final classifier by each of the two variable selection methods, for each of the four confounding levels, is shown in Figure 4(b). In Supporting Information S1, we show also the distribution of the *number* of variables that are selected as being optimal for each degree of confounding. When there is no confounding between the group and the batch, almost no variables that are associated with the batch effect will be selected with any of the variable selection methods. As the level of confounding increases, the fraction of batch-related genes that are included in the final classifier increases, most rapidly for the Wilcoxon variable selection. Recall that these genes are not truly associated with the group discrimination, and that in fact they do not hold up as good discrimination rules when applied to a data set without this specific batch effect (as illustrated in Figure 4(a)).

**Figure 5:**
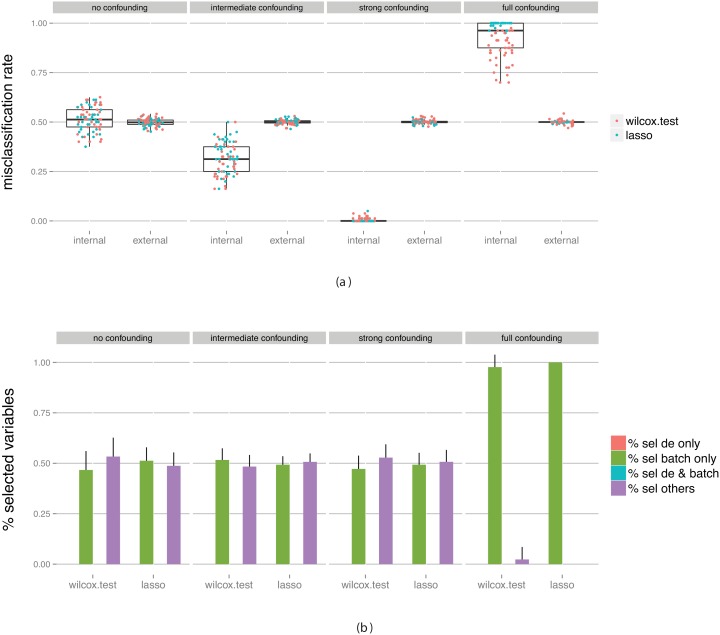
Evaluation of classifiers built on data without truly differentially expressed genes between the classes, but with a batch effect with various degree of confounding with the class labels, after the elimination of this batch effect with ComBat. (a) Estimated predictive performance from the outer cross-validation (internal) and obtained by applying the constructed classifier to an external test set (external). (b) The fraction of predictor variables selected for the final classifier that were simulated to be differentially expressed and/or associated with the batch. The bars summarize results across all classifiers and all data set replicates. The bar heights represent the average fraction of variables extracted from each category, and the error bars extend one standard deviation above the average. Note that since there are no truly differentially expressed genes in this data set the height of the two corresponding bars is zero.

Next, we assume that we are indeed aware of the existence of the batch effect (for example, we may know that the samples were processed at different times or come from different data sets), and we apply ComBat [23] to the entire training data set (that is, before the outer cross-validation is applied) in order to eliminate the effect of the batch before training the classifier and performing the cross-validation to estimate its performance. The resulting performance estimates are shown in Figure 5(a). We note that in the absence of truly differentially expressed genes, and with intermediate or strong confounding, the batch effect removal clearly fails to eliminate the bias in the performance estimates. The cross-validation still overestimates the true performance (the ‘internal’ estimate is systematically lower than the ‘external’ value). This is likely attributable to the confounding between the group and batch factors, which affects the batch effect removal. More precisely, when we perform the batch effect removal, we use the group factor as a covariate for ComBat, essentially asking the method to retain the information that can be associated with the group factor. If the batch effect and the group factor are partly confounded, this implies that ComBat may not eliminate the full impact of the batch factor, and the part of the batch-related signal that is associated with the group factor may be retained also after the batch effect removal. Of course, this signal is not seen in the external validation data set.

Since the batch effect is the only non-random effect in this data, eliminating it makes the previously batch-affected variables indistinguishable from the rest of the variables. Thus, it is not surprising that around half of the selected variables are “batch-related”, and the other half are “non-batch-related” (Figure 5(b)).

An interesting effect is seen in the right-most panel of the figure. When the batch is completely confounded with the group labels, and the batch effect is removed using ComBat, the cross-validation often gravely underestimates the classification performance. This can be explained by an overcompensation mechanism. In this case, since the batch and the group factor are in fact identical, we can not provide the group factor as a covariate to ComBat in order to retain the information related to it. Instead, we just attempt to eliminate the effect of the batch on the expression data. In principle, one goal of the batch effect removal is to make the average expression of each gene equal in the two batches. Since the batch effect removal was performed on the entire training data set this means that if we, in the classifier construction, find a gene that is higher in group 2 than in group 1 in an outer training set, it necessarily has the opposite pattern in the corresponding outer test set. Hence, when we apply the trained classifier to this test set to estimate the performance, many of the samples will be assigned to the wrong group and the estimate of the misclassification rate will be high. However, for the external data set used to validate the classifier, these genes are equally distributed in the two groups and thus the classifier performs no better (or worse) than chance.

### Simulation study, alternative case

Here, we consider the case where, in contrast to the example above, there actually are some genes that are differentially expressed between the two groups that we wish to distinguish. As for the null case above, we examine the effect of a confounding variable (the batch), with varying degree of confounding between the batch and the group labels. In this case, since there are genes that are truly differentially expressed between the two groups of interest, we expect that it should be possible to obtain a classifier with good classification performance. However, if the batch effect is strong and confounded with the factor of interest, we anticipate that the variable selection may be guided towards these variables, which are not truly differentially expressed between the two populations and thus do not generalize well to independent data sets.

**Figure 6:**
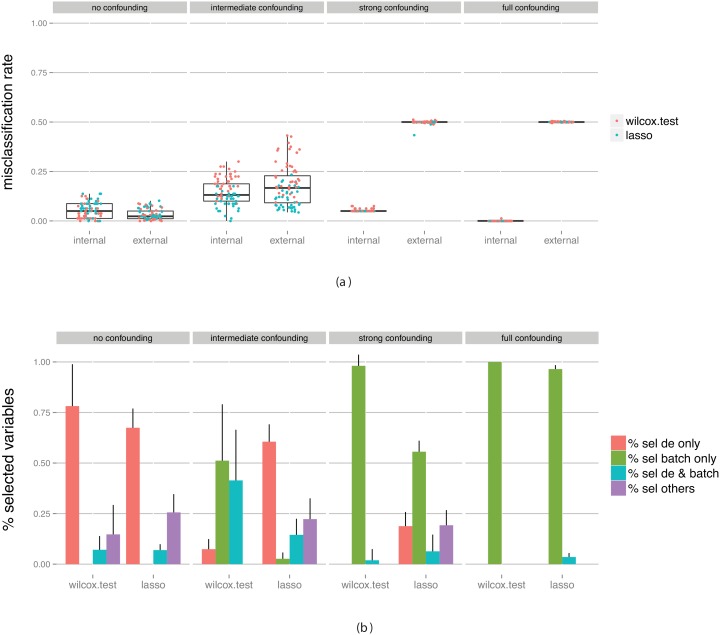
Evaluation of classifiers built on data containing truly differentially expressed genes between the classes, as well as a batch effect with various degree of confounding with the class labels. (a) Estimated predictive performance from the outer cross-validation (internal) and obtained by applying the constructed classifier to an external test set (external). (b) The fraction of predictor variables selected for the final classifier that were simulated to be differentially expressed and/or associated with the batch. The bars summarize results across all classifiers and all data set replicates. The bar heights represent the average fraction of variables extracted from each category, and the error bars extend one standard deviation above the average.

Figure 6(a) shows the performance for varying degree of confounding, when we made no attempt to eliminate the confounding factor. For low levels of confounding (the two left-most panels) there is no discernible bias in the cross-validation estimates. Moreover, we note that the misclassification rate is lower than in Figure 4(a), both for the training and the validation data set, thanks to the presence of some genes that are truly differentially expressed between the groups. We can see the effect also in Figure 6(b), which shows the fraction of the selected genes that were simulated to be truly discriminating and/or associated with the batch effect. For low levels of confounding, most of the genes selected by the lasso are indeed truly differentially expressed between the two groups. With the Wilcoxon test, many batch related variables are selected, and consequently the resulting classifiers perform worse (Figure 6(a)).

As the degree of confounding increases, the classifier tends to a higher extent to select genes that are associated with the batch effect, which provides a classifier that works well for the training data but that does not generalize well, especially for the Wilcoxon variable selection.

**Figure 7:**
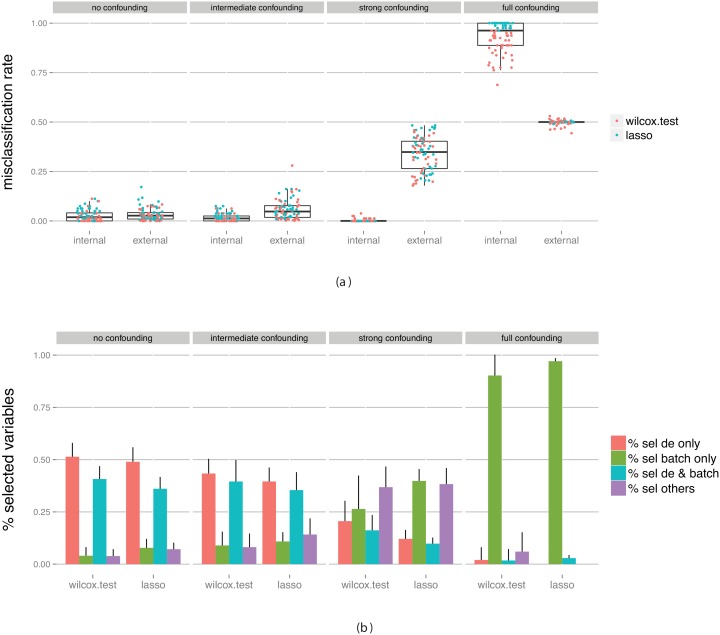
Evaluation of classifiers built on data containing truly differentially expressed genes between the classes, as well as a batch effect with various degree of confounding with the class labels, after the elimination of this batch effect with ComBat. (a) Estimated predictive performance from the outer cross-validation (internal) and obtained by applying the constructed classifier to an external test set (external). (b) The fraction of predictor variables selected for the final classifier that were simulated to be differentially expressed and/or associated with the batch. The bars summarize results across all classifiers and all data set replicates. The bar heights represent the average fraction of variables extracted from each category, and the error bars extend one standard deviation above the average.

Finally, we investigate the results obtained after eliminating the batch effect using ComBat (Figure 7). Comparing to Figure 6, we notice that if the degree of confounding is not too large, eliminating the confounding variable gives a better classifier (with lower misclassification rate), and a higher fraction of selected genes that are truly differentially expressed between the groups. Moreover, the bias in the cross-validation performance estimates is negligible.

**Figure 8:**
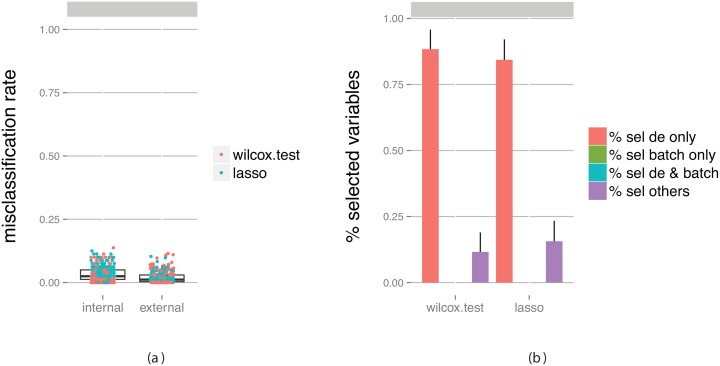
Evaluation of classifiers built on data containing truly differentially expressed genes between the classes, but no batch effect. (a) Estimated predictive performance from the outer cross-validation (internal) and obtained by applying the constructed classifier to an external test set (external). (b) The fraction of predictor variables selected for the final classifier that were simulated to be differentially expressed and/or associated with the batch. The bars summarize results across all classifiers and all data set replicates. The bar heights represent the average fraction of variables extracted from each category, and the error bars extend one standard deviation above the average. Note that since there is no batch effect in this data set the height of the two corresponding bars is zero.

For extensive confounding we make the same observation as for the null case: the elimination is not fully efficient and the performance estimates from the cross-validation are heavily biased. Moreover, for the full confounding case we again overcompensate in the batch effect elimination, and thus the cross-validation underestimates the classification performance. The results can be compared to those given in Figure 8, which shows the performance measures obtained from a data set without any added batch effect. This represents the situation that we would like to achieve with the batch effect elimination. In this case, the classification accuracy is good, and the bias of the cross-validation estimate is low. The results are similar to those obtained with a low degree of confounding, which suggests that batch effects can be removed if they are not too heavily confounded with the variable of interest. This stresses the importance of good experimental design or careful merging of data sets, in order to avoid confounding as much as possible. It also highlights the importance of not blindly trusting reported cross-validation based performance estimates, since they may be heavily biased in the presence of (perhaps hidden) confounders.

## Discussion

The increasing amount of publicly available gene expression data provides researchers with the potential of combining them in order to create large collections of samples, which may provide higher power in addressing research hypotheses. Many researchers have already taken advantage of the large collection of public data, either by considering exclusively already published data sets, or by combining the public data with their own generated data [39], [40]. When combining data from different studies, it is important to be aware that the data has typically been collected in different places, with different equipment and under different external conditions. All these factors are likely to have considerable effects on the measured expression levels, referred to as “batch effects”. As individual studies grow larger and larger, it is increasingly likely that also samples within the same data set have been analyzed at different time points and under different conditions, and may thus be subject to the same type of unwanted variation as data from different studies. Moreover, study dropout and unknown confounders can introduce unwanted variation in otherwise well designed studies. Batch effects can be a big obstacle when combining data sets, and their characterization and potential elimination have recently received much attention in the literature (e.g., [7], [8], [23], [25]).

There are several, qualitatively different, approaches to combining a collection of data sets, depending on the research question of interest. One widely used approach is meta-analysis, which aggregates the results obtained by performing the analysis of interest separately on each data set. Meta-analysis is appropriate for example to examine whether a certain gene is consistently found to be associated with a given phenotype across several studies. However, meta-analysis is not always a feasible alternative. For example, to perform unsupervised analysis like clustering, or to construct a classifier, the different data matrices typically have to be merged in order for the analysis to profit from the increased sample size. In these situations, unaddressed batch effects may severely compromise the results of the analysis by concealing the real signal or introducing an artificial one [7]. In this paper we have discussed the performance evaluation of classifiers via cross-validation, and how the performance estimates are affected by the presence of batch effects in the training data, with various degree of confounding between the batch variable and the main grouping variable. The focus on estimation bias (in relation to the performance we would expect from the classifier built on the entire data set and applied to an external test set) is a main difference between this study and previous studies, which have mostly focused on the predictive performance and how it relates to the presence of batch effects [11]. We have shown that in the presence of a batch effect with at least moderate level of confounding with the main grouping variable, the performance estimates obtained by cross-validation are highly biased. This stresses the importance of careful consideration of potential confounding effects when merging data sets from different studies or when samples within the same study have to be processed at different time points or under otherwise different external conditions. The presence of a batch effect that was completely non-confounded with the signal of interest did not introduce bias in the performance estimates obtained by cross-validation.

Unless the batch effect is heavily confounded with the outcome of interest, eliminating the batch effect typically improves the performance of the resulting classifier. From this point of view, hence, elimination of batch effects before constructing a classifier is beneficial. However, the bias in the cross-validation performance estimates is not eliminated by the batch effect removal, and consequently the cross-validation performance estimates obtained after batch effect elimination are not more reliable measures of the true performance than those obtained without batch effect elimination. This apparent insufficiency of the batch effect removal stresses the importance of careful experimental design where confounding between potential batch effects and the signal of interest are avoided as much as possible. In other words, batch effect removal methods should not be trusted blindly as a ‘post-experimental’ way of rescuing a badly designed experiment.

The presented results have implications not only for binary classifier evaluation, but also for evaluation of multi-class classifiers and predictive models with other endpoints, such as survival time. Moreover, the results presented here are important for differential expression analysis and other approaches for ranking genes in terms of significance. We have shown that in the presence of batch effects that are confounded with the signal of interest, many of the highly ranked variables are associated only with the batch effect and not truly differentially expressed between the interesting groups. Consequently, they are unlikely to hold up as statistically, as well as biologically, significant discriminators in any other study. For the two variable selection methods we evaluated, the Wilcoxon test seemed to be more sensitive to the confounding, and included more batch effect related genes in the final classifier than the lasso variable selection. We hypothesize that one reason for this is that the lasso considers all variables simultaneously when doing the selection, and tends to include relatively uncorrelated variables in the final selection [41]. The Wilcoxon test, on the other hand, is applied to the genes independently, and highly correlated genes are likely to obtain similar ranking scores.

While the present study is focused on cross-validation as the method for estimating classification performance, other methods have been suggested for the same purpose (e.g., various bootstrap procedures or permutation tests). It has been shown that in general, the bias of these methods can be quite different [42]. However, this difference applies independently of the degree of confounding, and we expect the effect of the degree of confounding on the bias to be similar across the methods. Similarly, we expect the results to generalize to other batch effect removal methods and classifiers.

## Supporting Information

Supporting Information S1 **Additional results and simulation details.** Detailed description of the data simulation procedure, as well as further details and alternative representations of the reported results.
(PDF)

Supporting Information S2 **R code used for data simulation and analysis.** Documented R code for the simulation procedure as well as the analysis and result generation.
(HTML)
