# Supplementary material for: Batch Effect Confounding Leads to Strong Bias in Performance Estimates Obtained by Cross-Validation
Source: PLoS One. 2014 Jun 26;9(6):e100335. doi: 10.1371/journal.pone.0100335 (PMC4072626; doi:10.1371/journal.pone.0100335)
Supplement: Supporting Information S1 — Additional results and simulation details. Detailed description of the data simulation procedure, as well as further details and alternative representations of the reported results. (PDF) [file pone.0100335.s001.pdf]

Batch effect confounding leads to strong bias in performance estimates obtained by cross-validation

SUPPORTING INFORMATION

Charlotte Soneson[1,†,*], Sarah Gerster[1,†], Mauro Delorenzi[1,2,3]

1 SIB Swiss Institute of Bioinformatics, Lausanne, Switzerland

2 Ludwig Center for Cancer Research, University of Lausanne, Lausanne, Switzerland

3 Oncology Department, University of Lausanne, Lausanne, Switzerland

† these authors contributed equally to this work

∗ E-mail: charlotte.soneson@isb-sib.ch

A Data simulation procedure

A.1 Differentially expressed features

We simulate data for the *null* (no genes are differentially expressed) and the *alternative* ($\sim 1\%$ of the genes are differentially expressed) scenario. For each simulated data set, we sample the index of the genes to be differentially expressed from a uniform distribution. The sign of the differential expression is chosen randomly for each feature based on the sign of a $N(0, 1)$ variable.

A.2 Features affected by batch effect

While the simulated validation data sets are not affected by batch effect, we introduce this artifact in most training sets. In these cases, we select about half of the features to be affected by batch effect. For each simulated data set, we sample the index of the affected genes from a uniform distribution. The sign of the batch effect is chosen randomly for each feature based on the sign of a $N(0, 1)$ variable.

Note that the sampling of the features affected by differential expression and by batch effect are done independently. Hence, a gene can be both, differentially expressed and affected by batch effect.

A.3 Strength of the confounding between the effect of the outcome variable and the batch effect

We simulate the samples to belong to one of two groups (outcome variable): $g1$ or $g2$. Each sample was processed in one of the two batches: $b1$ or $b2$. The strength of the confounding between the group and the batch depends on how the samples are partitioned into the batches:

- no confounding means that half of the $g1$ samples are in $b1$ and the other half in $b2$; same for $g2$
- intermediate confounding means that 75% $g1$ samples are in $b1$ and 25% in $b2$; vice versa for $g2$
- strong confounding means that 95% $g1$ samples are in $b1$ and 5% in $b2$; vice versa for $g2$
- full confounding means that all $g1$ samples are in $b1$ and all $g2$ samples in $b2$

A.4 Outline of the data simulation procedure

The outline below summarizes the strategy followed to simulate the 10 replicate data sets for a given setting (e.g., *null* data affected by batch effect featuring intermediate confounding between the effect of the outcome variable and the batch effect). For details, please refer to the file `S2_Rcode_bias-cv.html` containing the documented R code used to simulate the data sets.

1. Select the set of indices for the features to be differentially expressed and/or affected by batch effect (see A.1).
2. Set the sign for the direction of the differential expression and/or batch effect for each feature (see A.2).
3. For each replicate:
   - Sample 680 samples (80 for the training and 600 for the validation data set) without replacement from the original data frame.
   - Split samples into categories according to the choice made for the strength of the confounding (see A.3):
     - $g1b1$: samples from group one ($g1$) in batch one ($b1$) in the training set. The number of assignments to this group depends on the choice for *confounding*. In a balanced case without confounding, 20 samples are assigned to this category.

- Analogous procedure for $g2b1$, $g1b2$ and $g2b2$.

- Validation sets are balanced and not affected by batch effect. We assign half of the validation samples to $g1v$ and the other half to $g2v$.
- Set the mean for each category to the same value by subtracting the group mean (by feature) and adding the overall mean (by feature).
- Add group effect ($g1$ vs $g2$, DE) and batch effect ($b1$ vs $b2$, BE) to each sample group as appropriate. The effect sizes are defined as follow:
  - effect size of DE: *min.effect + Exp(n, exp.rate)*
  - because not all samples are affected in the same way by the DE, we add some noise to DE (log-normal distribution)
  - effect size of BE: *min.effect + Exp(n, exp.rate)*

  The values of the constants are provided in Table 1 below.
- Add some noise ($N(0,\ 2)$) to all samples/features.

The R code used for the data simulation is provided in the file `S2_Rcode_bias-cv.html`. A summary of the settings and values of the constants is listed in Table 1. An overview of all generated data sets is provided in Figure 1.

Table 1. Summary of possible parameter settings to generate the simulated data sets. DE stands for differential expression (of some features). BE stands for batch effect (affecting some of the features).

	TRAINING	VALIDATION
# samples	80	600
balance	balanced	balanced
DE	no/yes	no/yes
% DE genes	1	1
BE	no/yes	no
% BE genes	50	0
DE-BE confounding	no/inter/strong/full	no
min.effect	5	5
exp.rate	1.5	1.5
replicates	10	10

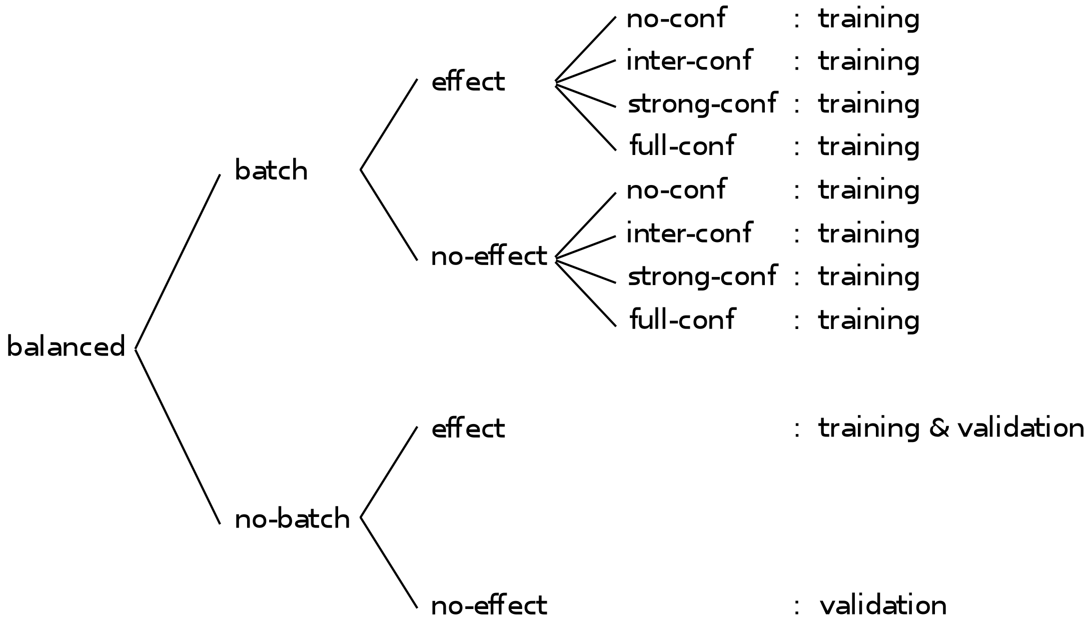

Figure 1. Overview of simulated data sets. Training sets were generated according to 9 different settings. For each setting, we simulated ten replicates. For external validation, we used simulated validation sets with balanced data and without batch effect.

A.5 Further settings for the data preparation

The study is performed on the first 10,000 features in each data set. Since the simulated effects are uniformly distributed on the original features, taking a subset will not alter the basic properties of the respective data set. The reduced number of features allows to save computation time, which was found to be better invested in processing several replicates for each data set.

B Selected hyperparameters and number of features

In this section we examine further the optimal classifiers built under different circumstances. In Table 2 we show the fraction of instances in which each of the possible hyperparameter values is selected as the optimal one, for each of the four classifiers. For the random forest, no hyperparameter tuning was performed. The tabulated hyperparameters are those selected in the cross-validation employed to build the final classifier, from the whole data set. Interestingly, all classifiers tend to select the optimal hyperparameter that leads to relatively robust methods (large k, small C, large λ), which are less prone to overfitting. One reason for this is likely that in the event of equal classification accuracy in the cross-validation, we select the hyperparameter leading to the least flexible model to avoid overfitting to the training data.

Table 2. The optimal hyperparameter chosen for each of the classifiers.

rfCMA		svmCMA		knnCMA		plrCMA	
nTrees	fraction	C	fraction	k	fraction	λ	fraction
500	1	10^{-5}	0.294	2	0	10^{-4}	0.0109
		0.001	0.419	5	0	0.001	0.00625
		0.1	0.224	8	0	0.01	0.0328
		1	0.0469	11	0	0.1	0.0844
		10	0.0156	15	1	1	0.141
						10	0.725

In Figure 2 we show the distribution of the optimal number of features selected by the two different variable selection approaches, summarized across all classifiers and confounding levels. With the Wilcoxon test, more variables were generally selected in data sets where there were truly differentially expressed genes than in the absence of such. For the lasso, the lack of differentially expressed genes led to a wider distribution of optimal feature subset sizes.

Figures 3 to 6 show similar figures, stratified further by the classifier. Similarly, Figures 7 to 10 show the results stratified by the degree of batch effect confounding.

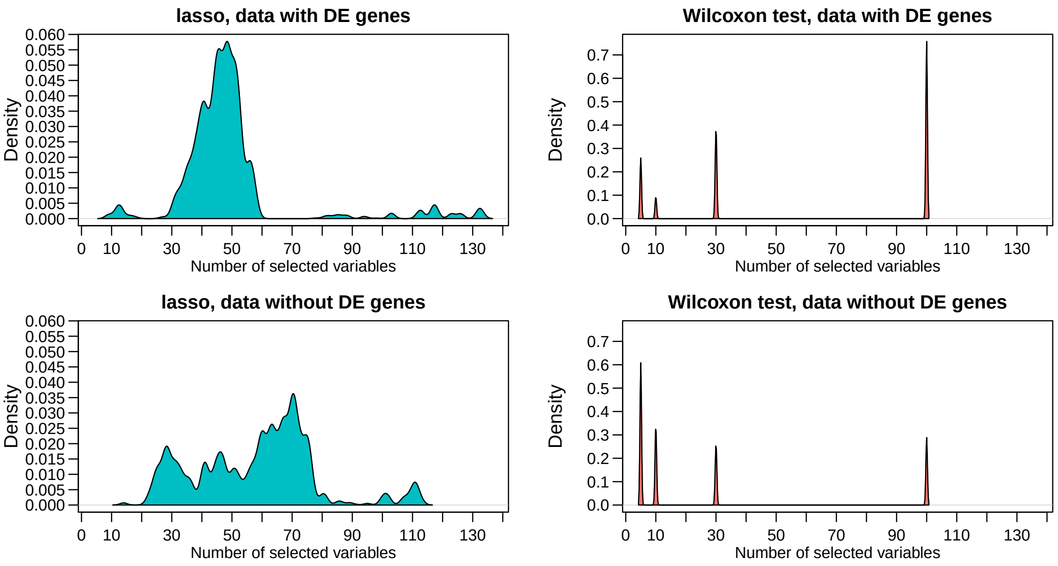

Figure 2. The distribution of the number of selected variables with each of the two variable selection methods (the lasso and the Wilcoxon test), in data sets with or without genes being truly differentially expressed between the two groups.

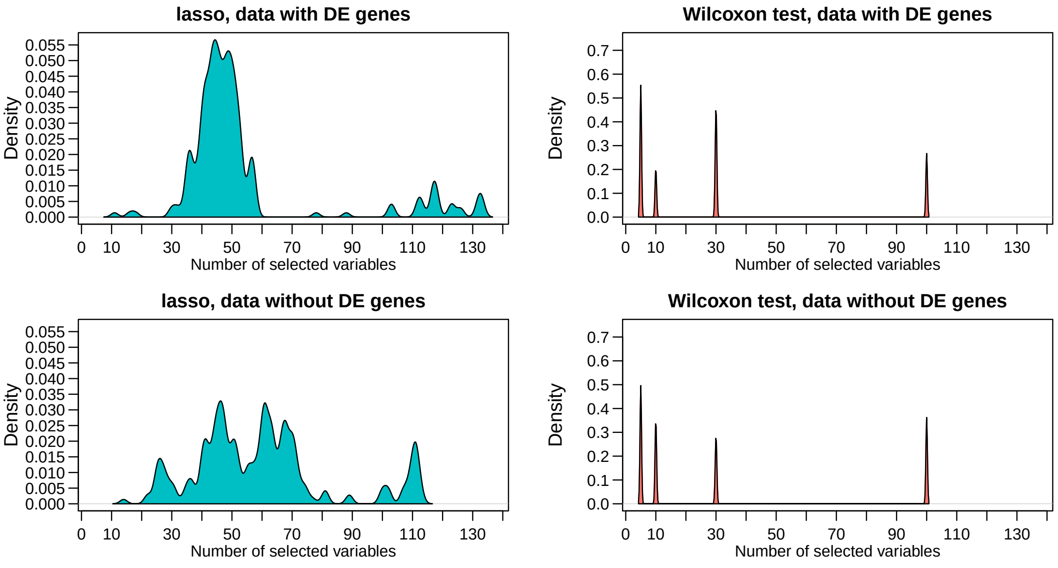

Figure 3. The distribution of the number of selected variables to include in the random forest classifier, for each of the two variable selection methods in data sets with or without genes being truly differentially expressed.

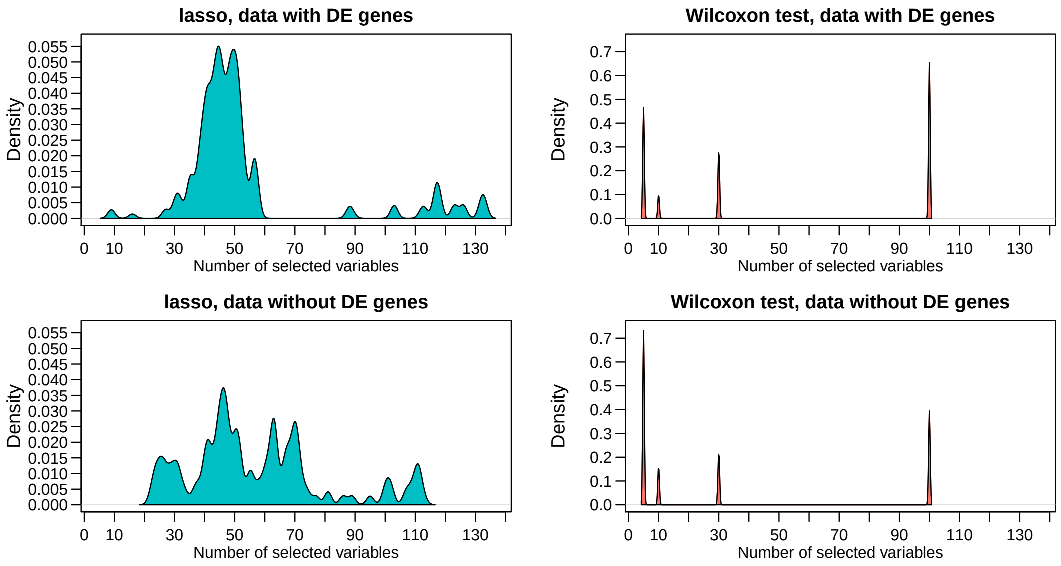

Figure 4. The distribution of the number of selected variables to include in the SVM classifier, for each of the two variable selection methods in data sets with or without genes being truly differentially expressed.

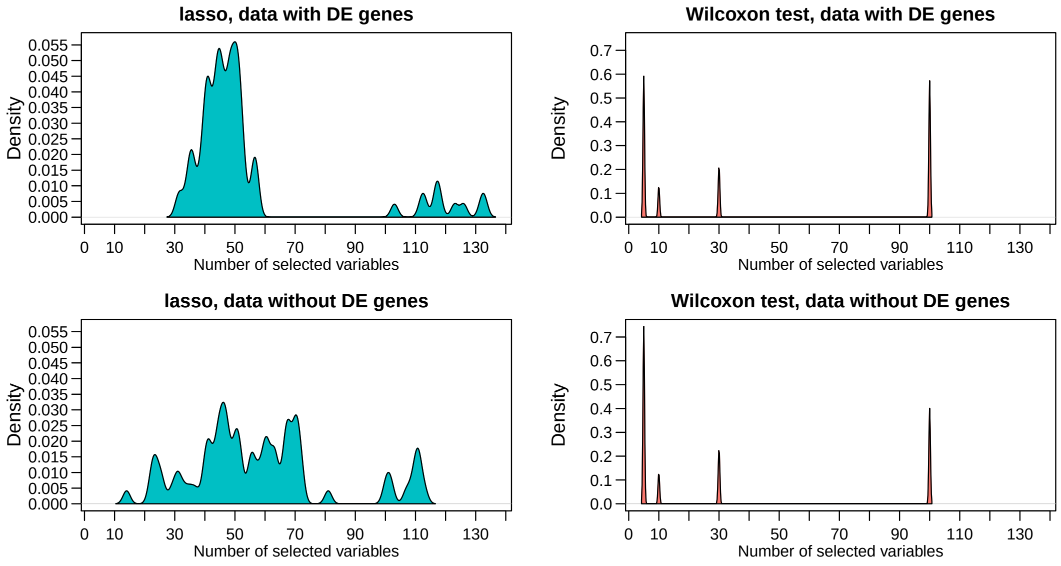

Figure 5. The distribution of the number of selected variables to include in the PLR classifier, for each of the two variable selection methods in data sets with or without genes being truly differentially expressed.

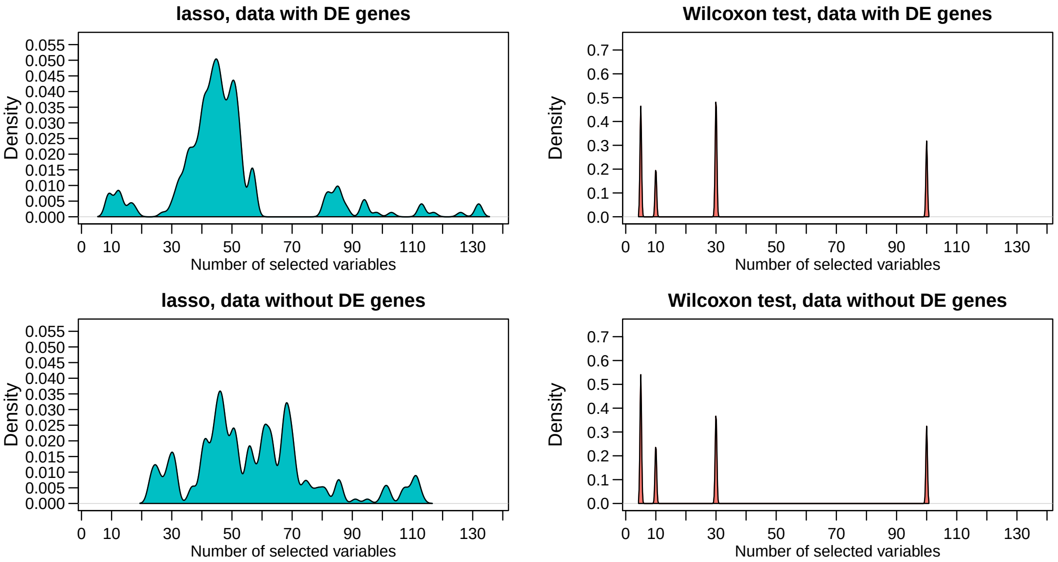

Figure 6. The distribution of the number of selected variables to include in the kNN classifier, for each of the two variable selection methods in data sets with or without genes being truly differentially expressed.

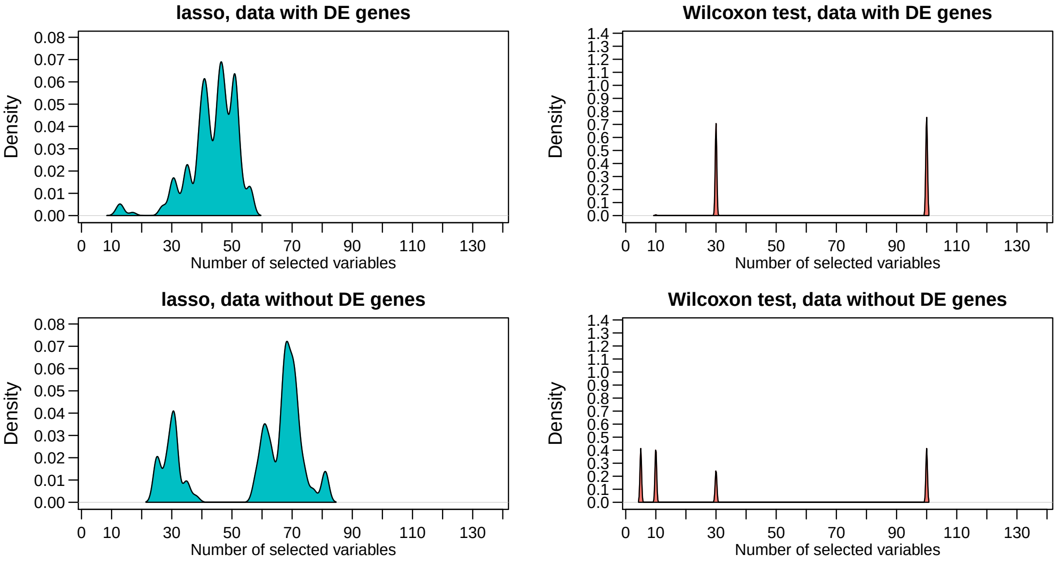

Figure 7. The distribution of the number of selected variables for each of the two variable selection methods in data sets with or without genes being truly differentially expressed, and without confounding with the batch effect.

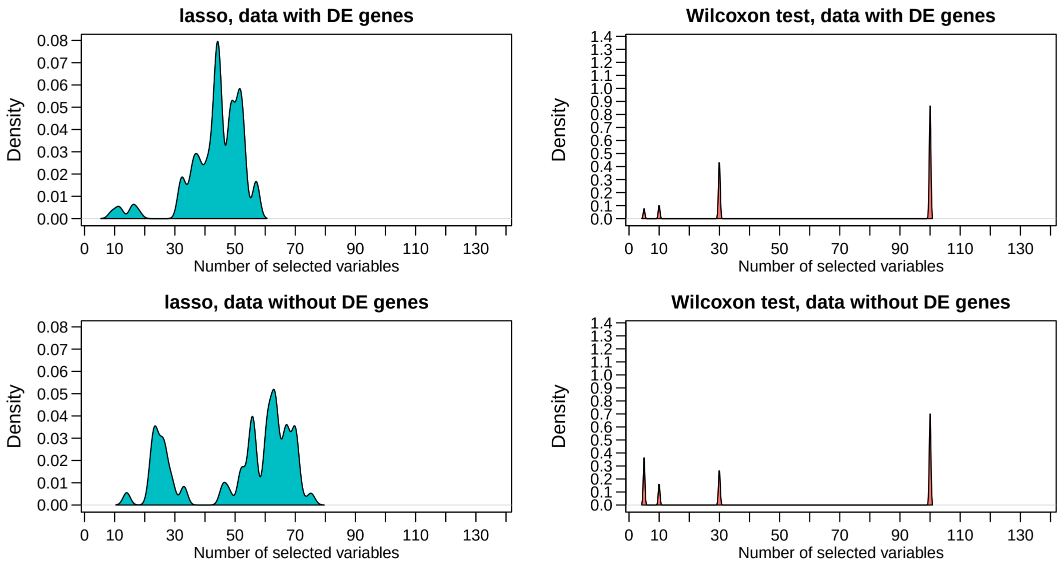

Figure 8. The distribution of the number of selected variables for each of the two variable selection methods in data sets with or without genes being truly differentially expressed, with intermediate confounding with the batch effect.

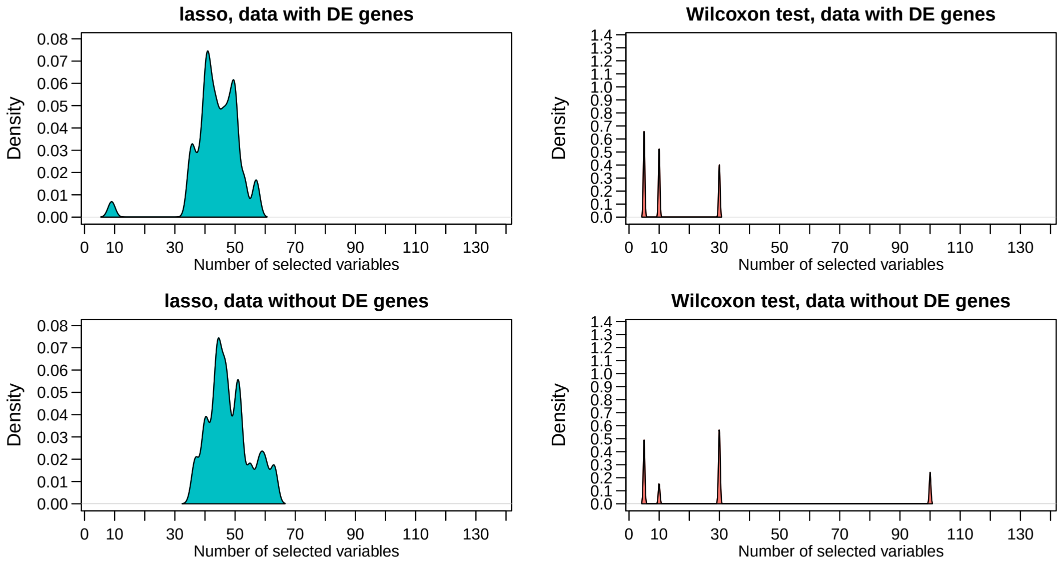

Figure 9. The distribution of the number of selected variables for each of the two variable selection methods in data sets with or without genes being truly differentially expressed, with almost full confounding with the batch effect.

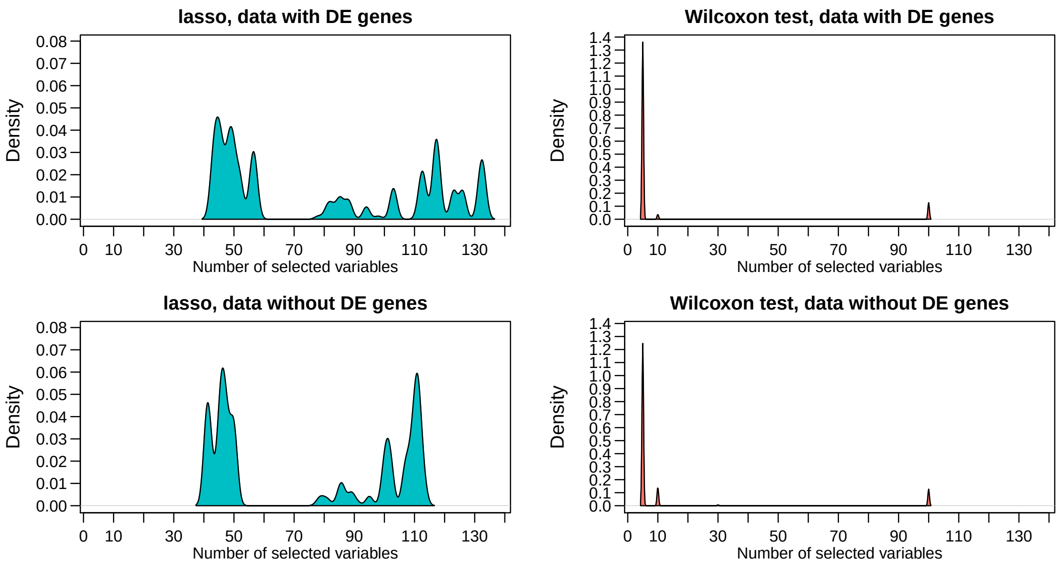

Figure 10. The distribution of the number of selected variables for each of the two variable selection methods in data sets with or without genes being truly differentially expressed, with full confounding with the batch effect.

C Other performance measures

In the main manuscript, we present the classification performance in terms of the misclassification rate. This usually works well if the groups are of equal size. Here, we present results with other performance measures.

C.1 Average of sensitivity and specificity

Instead of considering the overall classification performance, it is common to instead consider the class-specific performances of the classifier, and record the fraction of correctly classified samples in each class separately. In two-class classification problems, these class-specific performance measures are often referred to as the *sensitivity* (the fraction of correctly classified samples in the "positive" group) and the *specificity* (the fraction of correctly classified samples in the "negative" group). The average of the sensitivity and the specificity provides an alternative to the overall misclassification rate as a performance measure.

Figure 11 shows the average sensitivity and specificity for the null data set, before and after batch effect removal, respectively (compare to Figures 3(a) and 4(a) in the manuscript).

Figure 12 shows the average sensitivity and specificity for the alternative data set, before and after batch effect removal, respectively (compare to Figures 5(a) and 6(a) in the manuscript).

C.2 Area under the ROC curve

Some classification rules return a continuous score, which is used as the basis for the class label assignment. In the simplest two-class case, all samples with scores below a certain cutoff are assigned one label, and all samples with scores above the cutoff are assigned the other label. If the classifier returns a continuous score, we can evaluate its performance by means of a ROC curve, which depicts 1-specificity versus sensitivity obtained as the cutoff level varies throughout its range. The area under the ROC curve (often abbreviated AUC) provides a summary measure of the classifier's performance.

Figure 13 shows the AUC for the null data set, before and after batch effect removal, respectively (compare to Figures 3(a) and 4(a) in the manuscript).

Figure 14 shows the AUC for the alternative data set, before and after batch effect removal, respectively (compare to Figures 5(a) and 6(a) in the manuscript).

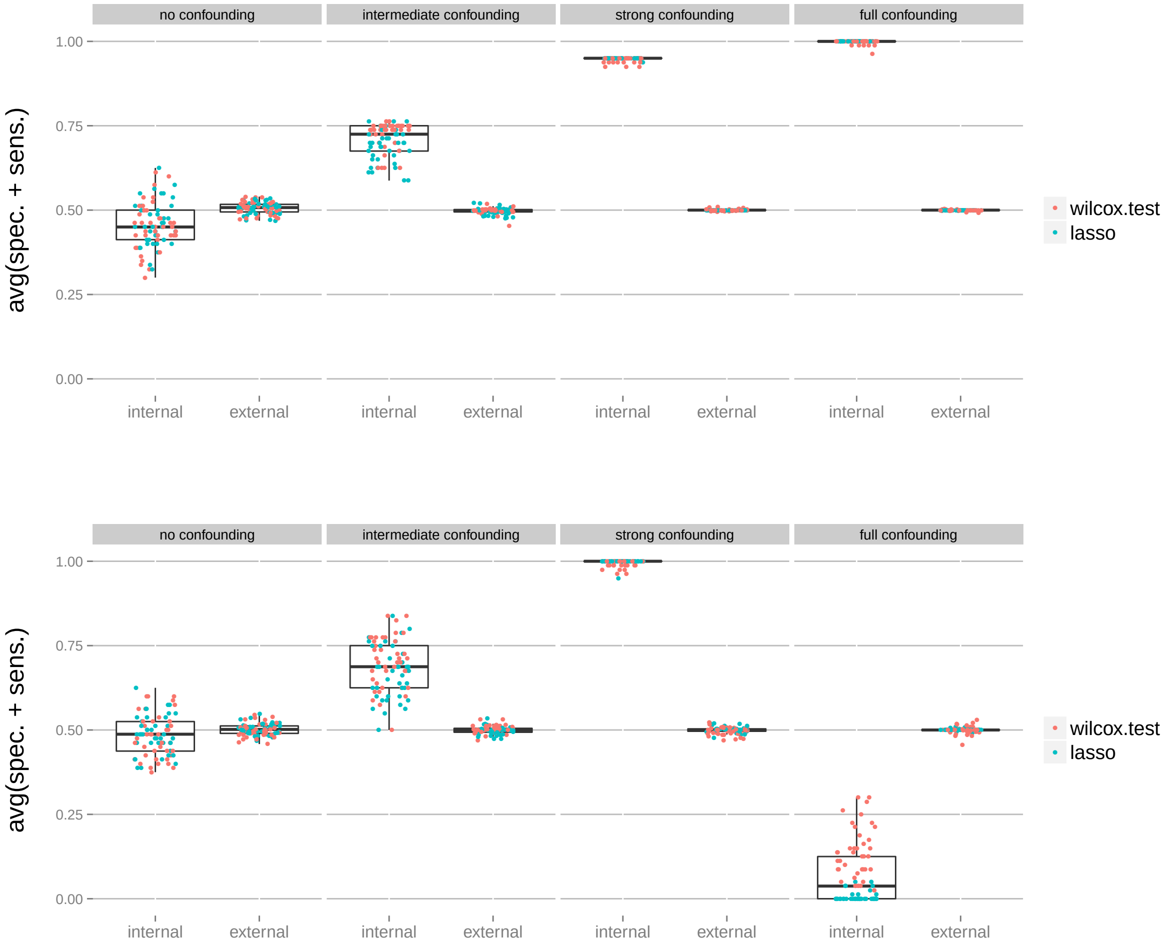

Figure 11. Evaluation of classifiers built on data without truly differentially expressed genes between the classes, as well as a batch effect with various degree of confounding with the class labels. (a) Estimated predictive performance from the outer cross-validation (internal) and obtained by applying the constructed classifier to an external test set (external), before the batch effect removal. (b) Estimated predictive performance from the outer cross-validation (internal) and obtained by applying the constructed classifier to an external test set (external), after the batch effect removal.

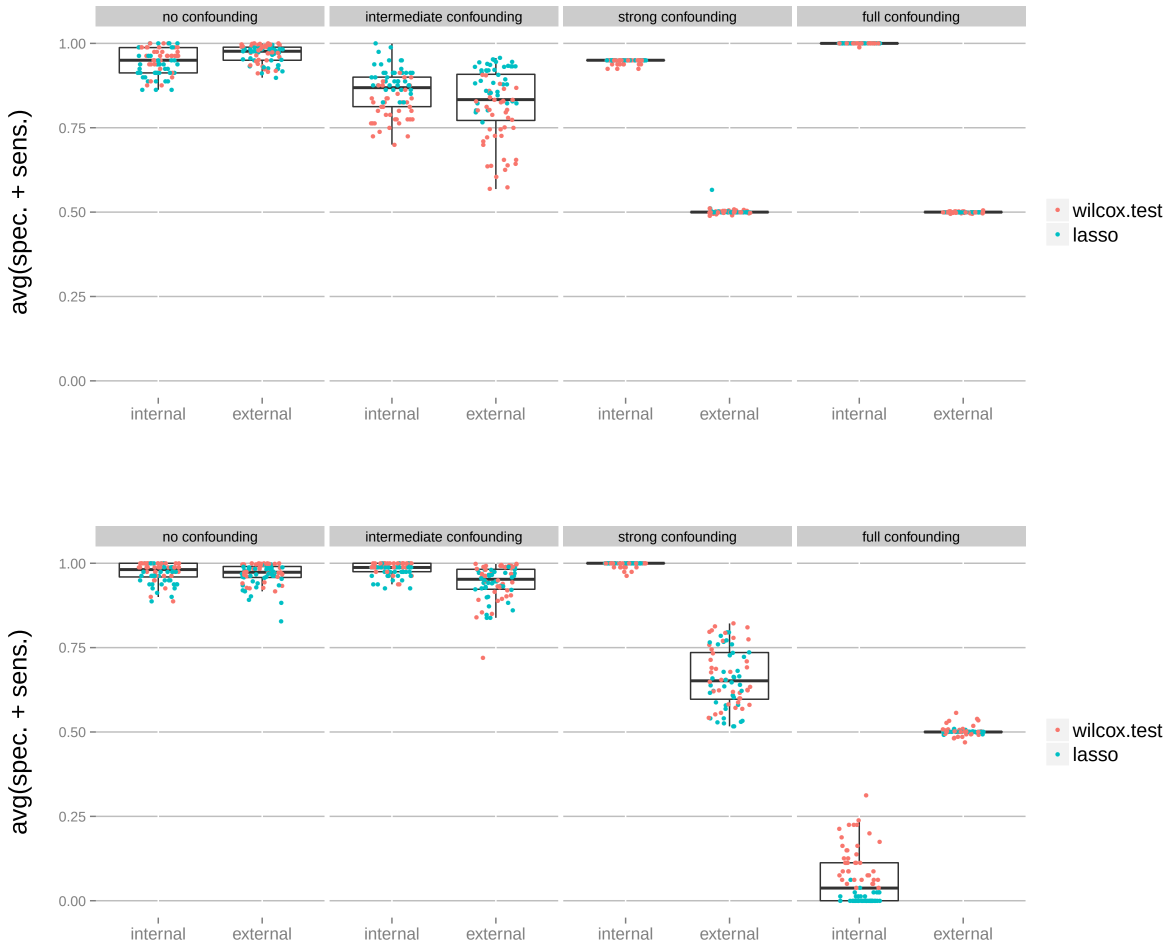

Figure 12. Evaluation of classifiers built on data containing truly differentially expressed genes between the classes, as well as a batch effect with various degree of confounding with the class labels. (a) Estimated predictive performance from the outer cross-validation (internal) and obtained by applying the constructed classifier to an external test set (external), before the batch effect removal. (b) Estimated predictive performance from the outer cross-validation (internal) and obtained by applying the constructed classifier to an external test set (external), after the batch effect removal.

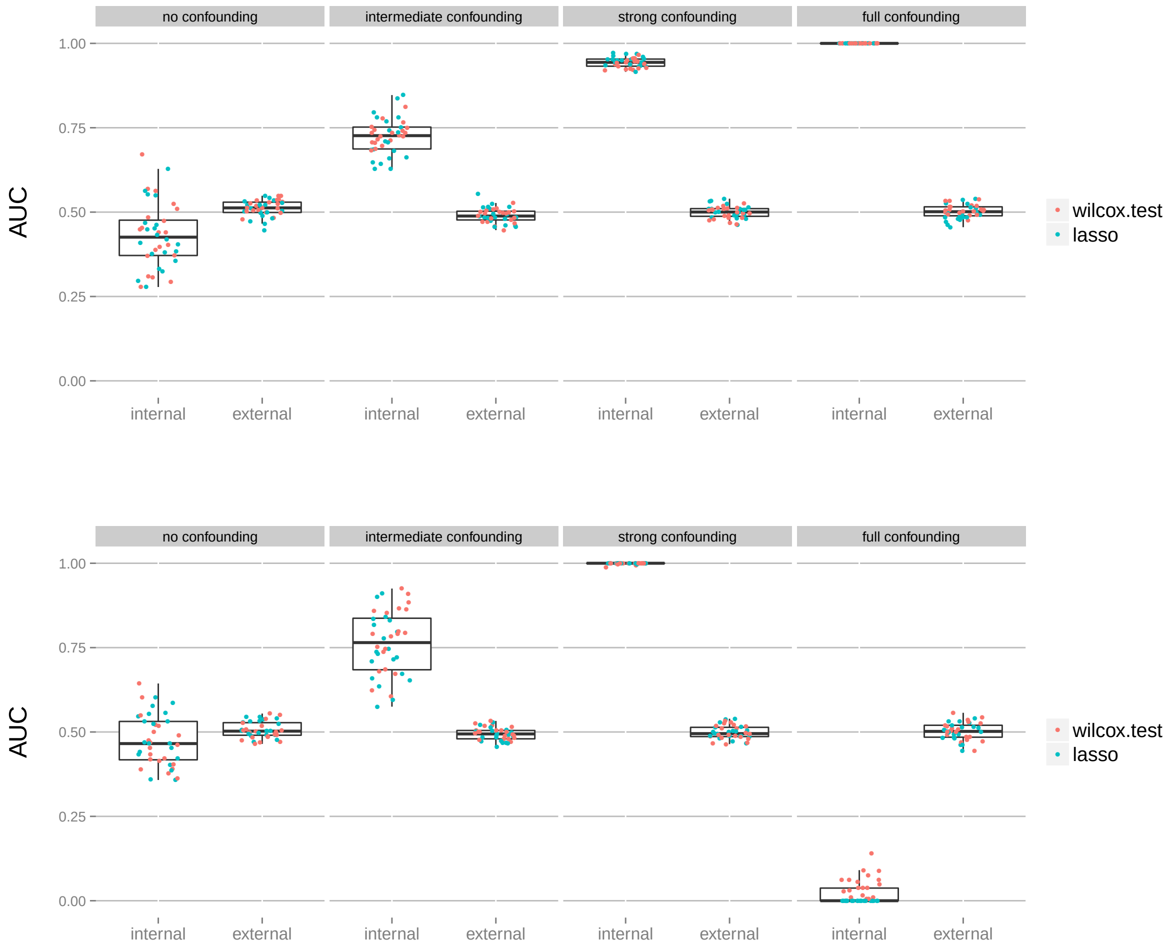

Figure 13. Evaluation of classifiers built on data without truly differentially expressed genes between the classes, as well as a batch effect with various degree of confounding with the class labels. (a) Estimated predictive performance from the outer cross-validation (internal) and obtained by applying the constructed classifier to an external test set (external), before the batch effect removal. (b) Estimated predictive performance from the outer cross-validation (internal) and obtained by applying the constructed classifier to an external test set (external), after the batch effect removal.

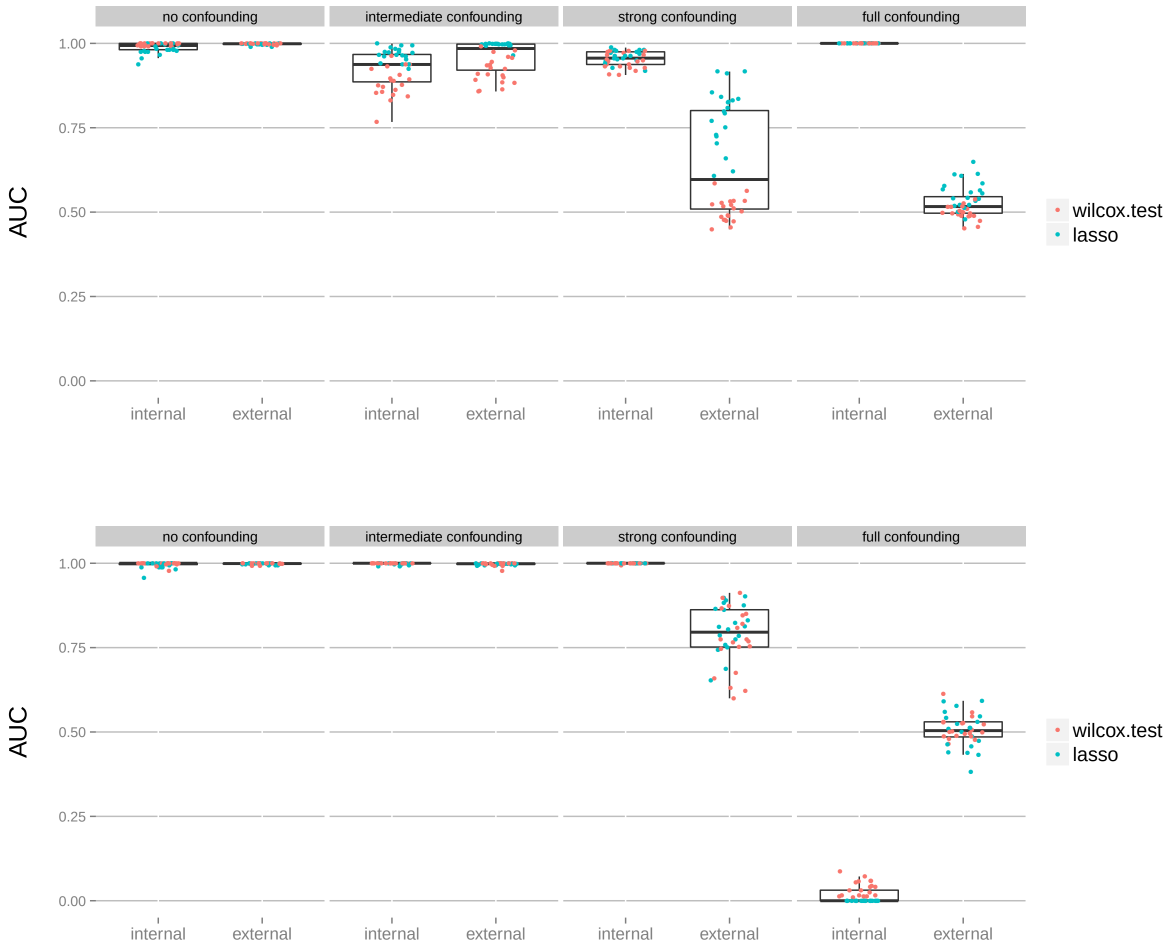

Figure 14. Evaluation of classifiers built on data containing truly differentially expressed genes between the classes, as well as a batch effect with various degree of confounding with the class labels. (a) Estimated predictive performance from the outer cross-validation (internal) and obtained by applying the constructed classifier to an external test set (external), before the batch effect removal. (b) Estimated predictive performance from the outer cross-validation (internal) and obtained by applying the constructed classifier to an external test set (external), after the batch effect removal.

D Different classifiers

In the main paper, all results were merged across the four classifiers that were used. Here, we break up the results for the different classifiers and show that they indeed perform very similarly on these data sets (Figures 15-16).

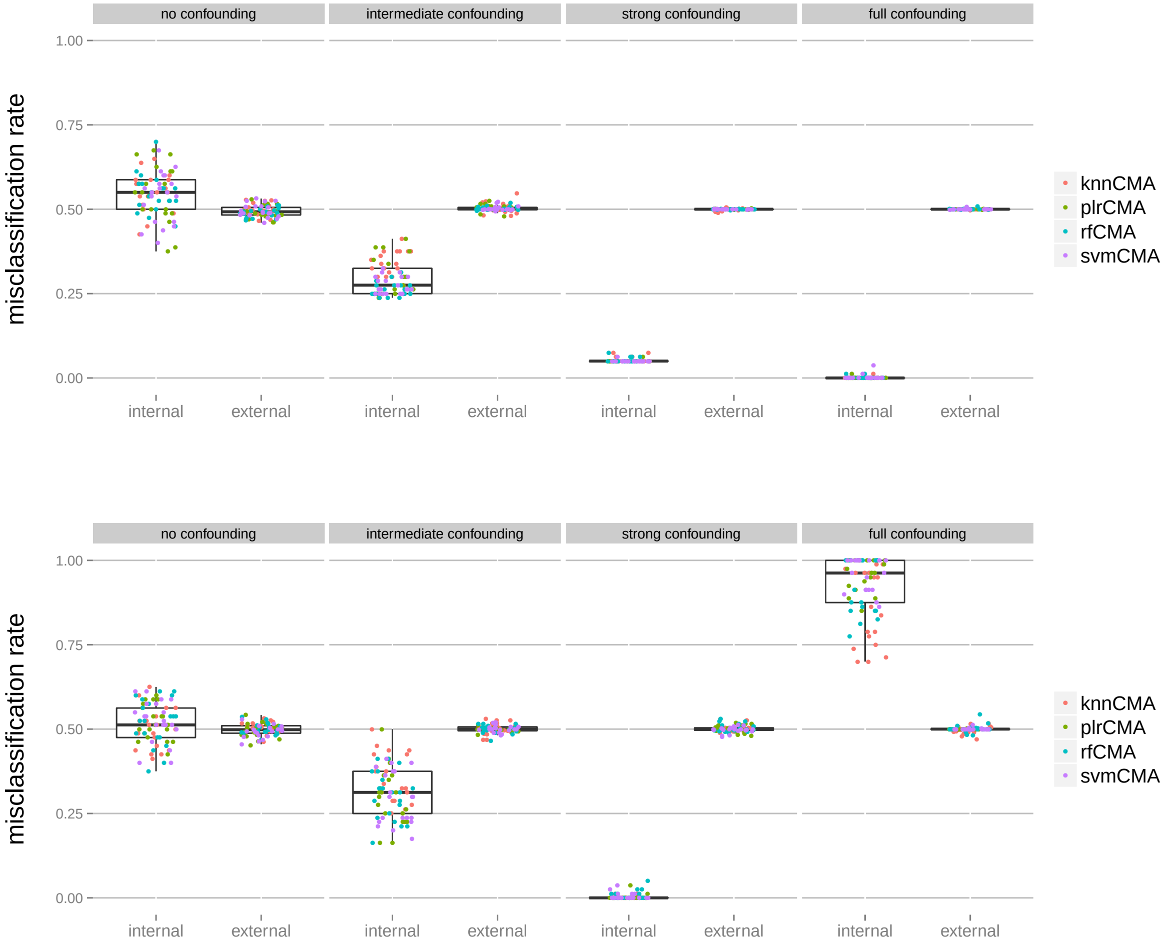

Figure 15. Evaluation of classifiers built on data without truly differentially expressed genes between the classes, as well as a batch effect with various degree of confounding with the class labels, colored by the classifier. (a) Estimated predictive performance from the outer cross-validation (internal) and obtained by applying the constructed classifier to an external test set (external), before the batch effect removal. (b) Estimated predictive performance from the outer cross-validation (internal) and obtained by applying the constructed classifier to an external test set (external), after the batch effect removal. Overall, the different classifiers performed similarly on these data sets.

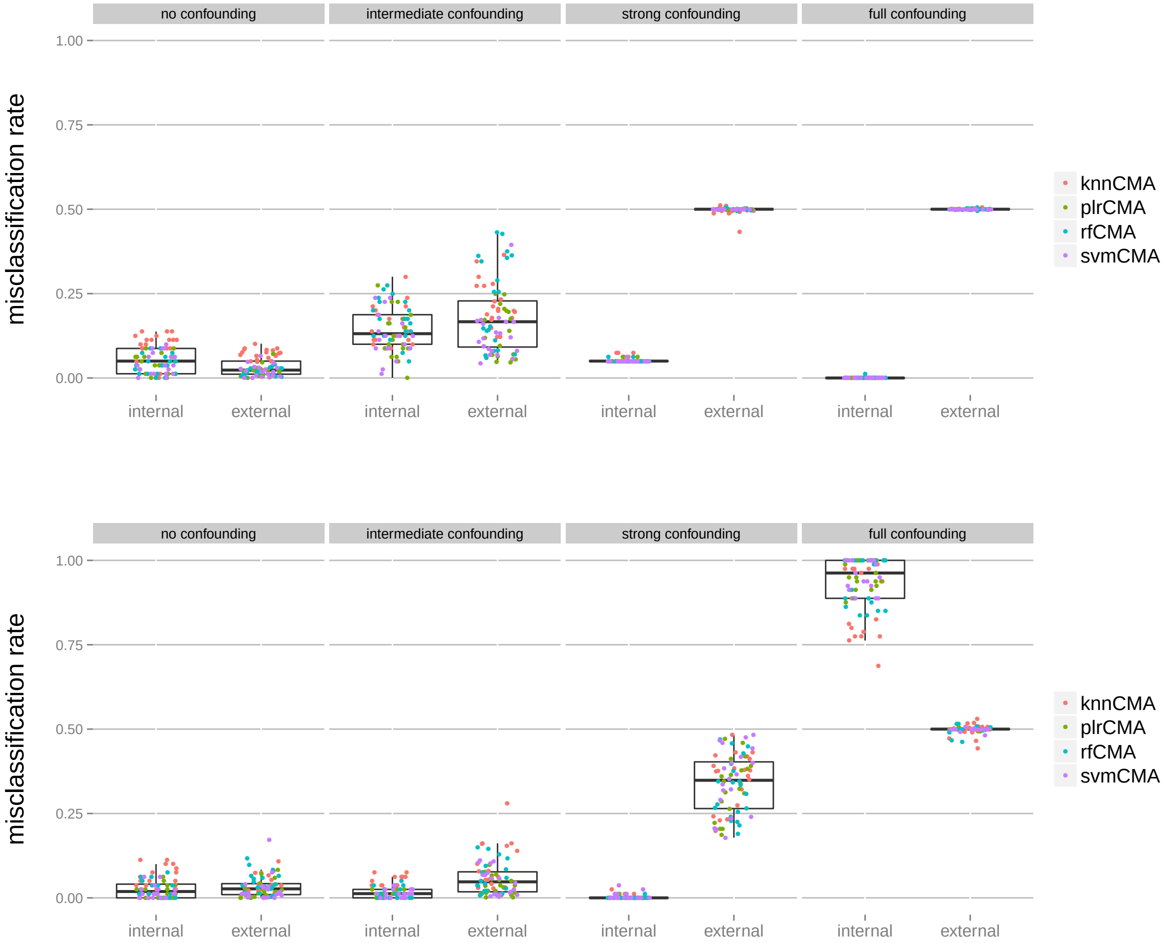

Figure 16. Evaluation of classifiers built on data containing truly differentially expressed genes between the classes, as well as a batch effect with various degree of confounding with the class labels, colored by the classifier. (a) Estimated predictive performance from the outer cross-validation (internal) and obtained by applying the constructed classifier to an external test set (external), before the batch effect removal. (b) Estimated predictive performance from the outer cross-validation (internal) and obtained by applying the constructed classifier to an external test set (external), after the batch effect removal. Overall, the different classifiers performed similarly on these data sets.
