# Supplementary material for: Batch Effect Confounding Leads to Strong Bias in Performance Estimates Obtained by Cross-Validation
Source: PLoS One. 2014 Jun 26;9(6):e100335. doi: 10.1371/journal.pone.0100335 (PMC4072626; doi:10.1371/journal.pone.0100335)
Supplement: Supporting Information S2 — R code used for data simulation and analysis. Documented R code for the simulation procedure as well as the analysis and result generation. (HTML) [file pone.0100335.s002.html]

R code for the manuscript "Batch effect confounding leads to strong bias in performance estimates obtained by cross-validation"


# R code for the manuscript "Batch effect confounding leads to strong bias in performance estimates obtained by cross-validation"

## Sarah Gerster, Charlotte Soneson, Mauro Delorenzi

### Tue Jan 21 13:10:45 2014

- Simulating the data
- Training classifiers
  - Settings for input data and classifier
  - Load simulated data corresponding to chosen settings
  - Remove the batch effect
  - Limit required computation time
  - Split the training data into sets for cross-validation
  - Nested CV scheme
  - Learn classifier to apply to external validation data sets
- External validation of classifiers
- Defined functions and (explicitly) loaded packages
  - Loaded R packages
  - Function to simulate the data
  - Altered definition of the knn function
  - Function to validate a classifier
  - Function to scale the data
  - Function to generate sample indices of CV sets
  - Function to generate a list of CV sets
  - Function to suppress some warnings
- R session information used to generate this document
- Makefile used for compilation of this document
- pandoc version used for conversion to html of this document


## Simulating the data

The procedure to simulate a data set is shown below with a concrete example.

First we set values for the different parameters:

```
# set strength and scope of effects for simulated data
# --> 1% of the features are differentially expressed (DE)
pde <- 0.01 
# --> 50% of the features are affected by batch effect (BE)
pb <- 0.5 
# --> set the minimal effect size for BE and DE
min_effect <- 5
# --> set rate for exponential distribution for DE and BE
exp_rate <- 1.5

# set a seed for reproducibility
set.seed(42)

# define the features that are affected by the two effect types
# --> indices of features affected by differential expression
de_indic <-  runif(ncol(X)) < pde
# --> indices of probesets affected by batch effect
batch_indic <- runif(ncol(X)) < pb
# --> sign of the differential expression
de_sign <- sign(rnorm(ncol(X)))
# --> sign of the batch effect
batch_sign <- sign(rnorm(ncol(X)))
```

We are now ready to call the function to simulate a new data set. As an example, we simulate a balanced data set with 80 samples in the training set and 600 in the test set:

```
# simulate a data set
sim_data <- sim.real(X, g1b1 = 20, g2b1 = 20, g1b2 = 20, g2b2 = 20,
                     g1v = 300, g2v = 300,
                     batch = "same", min.effect = min_effect, replace = FALSE, 
                     de.indic = de_indic, de.sign = de_sign,
                     batch.indic = batch_indic, batch.sign = batch_sign,
                     logarithmize = FALSE, exp.rate = exp_rate)
X_sim <- sim_data[["X.sim"]]
```

where X is the gene expression matrix from a real data set and the function sim.real() is defined in Defined functions and (explicitly) loaded packages. Parameters such as balance of the samples, or confounding between outcome and batch effect are controlled by specific choices for *g1b1*, *g2b1*, *g1b2* and *g2b2*.

## Training classifiers

A list of required packages and all own function definitions are provided in section Defined functions and (explicitly) loaded packages.

### Settings for input data and classifier

As an example, we provide the settings used to learn a classifier on one replicate of a balanced data set with 80 samples in the training set. It contains truly differentially expressed genes, a batch effect of the same magnitude as the differential expression and intermediate confounding between outcome and batches.

```
# set paths where data should be read and results stored
dataPath <- "data/"
resultPath <- "results/"

# set choices for data sets
nSamples <- 80 
nReplicate <- 1
balance <- "balanced"
diffExpression <- "effect"
batchEffect <- "same"
confounding <- "intermediate"
```

```
# settings for the corss-validation folds
# --> nested CV scheme => specify folds for both levels
cvFoldOuter <- 5
cvFoldInner <- 4

Additional settings are needed for the classifier itself. In the
example here, we consider a support vector machine (SVM) with feature
selection performed by a Wilcoxon test.
```

```
# set choices for variable selection
featureSelectionMethod <- "wilcox.test"       # wilcoxon test or lasso
nbFeaturesOnArray <- 10000                    # How many of the 61528
                                              # features should be used
                                              # for the analysis? 
nbFeatures.vect <- c(5, 10, 30, 100)          # NOTE: values in increasing
                                              #       order
normFraction.vect <- 1                        # NOTE: values in decreasing
                                              #       order

# set choices for classifier
classifier <- "svmCMA"
tuning.vals <- list(cost = c(1e-5, 1e-3, 0.1, 1, 10))
favorSmallModel <- "min"                # in case of ex-aequo performance,
                                        # select model with min/max
                                        # tuning value (depending on which
                                        # one leads to a smaller model)

# further settings for the data handling
# --> stratified CVs
stratifiedCVsplit <- TRUE
# --> batch effect removal
batchEffectRemoval <- TRUE
# --> scaling
scaling.method <- "zscore"
# --> verbosity (true =  additional output)
verbose <- FALSE
```

### Load simulated data corresponding to chosen settings

```
# load simulated data
load(file = paste(dataPath, "data/n", nSamples, "/", balance, "/",
       diffExpression, "/", batchEffect, "Batch/", confounding,
       "Confounding/replicate_", nReplicate, ".rdata", sep = ""))
# --> extract expression matrix
X <- sim_data[["X.sim"]]
# --> extract sample classes (g1 = control; g2 = case)
sampleType <- factor(sapply(colnames(X), function(x) substring(x, 1, 2)),
                     levels = c("g1", "g2"))
# --> extract simulated effects for differentially expressed genes
de.effect <- sim_data[["de.effect"]]
# --> extract simulated effects for genes subject to batch effect
batch.effect <- sim_data[["batch.effect"]]
# --> extract validation expression matrices and sample classes
X.val.effect <- sim_data[["X.val"]]
X.val.noEffect <- X.val.effect
X.val.noEffect[, sapply(colnames(X.val.noEffect),
                        function(x) substring(x, 1, 3)) == "g2v"] <-
  X.val.noEffect[, sapply(colnames(X.val.noEffect),
                          function(x) substring(x, 1, 3)) == "g2v"] - de.effect
sampleType.val <- factor(sapply(colnames(X.val.effect),
                                function(x) substring(x, 1, 2)),
                         levels = c("g1", "g2"))
```

### Remove the batch effect

When the batch effect is to be removed, we do it with the following code chunk:

```
if (confounding != "full") {
  mod <- model.matrix(~as.factor(substr(colnames(X), 1, 2)))
} else {
  mod <- NULL
}
X.bc <-
  ComBat(X, batch = sapply(colnames(X), function(x) substr(x, 3, 4)),
         mod = mod, numCovs = NULL, par.prior = TRUE, prior.plots = FALSE)
```

If there is no batch effect, or if we do not want to remove it, we set

```
X.bc <- X
```

### Limit required computation time

We only worked on a subset of the simulated features to save some computation time:

```
X.bc <- X.bc[1:nbFeaturesOnArray, ]
X.val.effect <- X.val.effect[1:nbFeaturesOnArray, ]
X.val.noEffect <- X.val.noEffect[1:nbFeaturesOnArray, ]
```

### Split the training data into sets for cross-validation

This is the split in outer CV sets. Hence, the sets that will be used to estimate the performance of the classifier.

```
set.seed(42)
cvSet.list <- get.cvIndexSets(nrCVfolds = cvFoldOuter,
                              sampleNames = colnames(X.bc),
                              stratified = stratifiedCVsplit)
```

### Nested CV scheme

Core part of the simulation implemented with a nested loop. The inner loop is used to tune the hyperparameters and number of features for the "optimal" classifier. The outer loop (on the outer cross-validation sets) is used to assess the classifier's performance.

```
# intialize data frame to store parameters and performance for a classifier
cvValidation.df <- 
    as.data.frame(matrix(NA, nrow = length(cvSet.list), ncol = 9,
                         dimnames = list(names(cvSet.list),
                             c("nbFeatures", "normFraction", "selectedFeatures",
                               "optimalParameter", "specificity", "sensitivity",
                               "misclassification", "average.prob", "auc"))))
cvSetCounter <- 0
# outer CV loop
for (cvSet in cvSet.list) {
  # keep track of which training set is being processed
  cvSetCounter <- cvSetCounter + 1
  if (verbose) {
    cat("PROCESSING CV SET", cvSetCounter, "OUT OF",
        length(cvSet.list), "\n")
  }
  # format data for usage with CMA package
  trainY <- sampleType[cvSet[["trainSampleIndic"]]]
  trainX <- t(X.bc[, cvSet[["trainSampleIndic"]]])  

  # initialize data frame to store settings/results from inner CV runs 
  tuningRes.df <-
      as.data.frame(matrix(NA, nrow = length(nbFeatures.vect) *
                           length(normFraction.vect) * length(tuning.vals[[1]]),
                           ncol = 3 + cvFoldInner,
                           dimnames = list(NULL, c("nbFeatures", "normFraction",
                                  "parameter", paste(rep("errInner", cvFoldInner),
                                                     1:cvFoldInner, sep = "")))))
  inner.res.counter <- 0
  # loop over grid of possible number of selected features (wilcoxon test)
  for (nbFeatures in nbFeatures.vect) {   
    if (verbose) {
        cat("   - TUNING HYPERPARAMETER WITH nbFeatures SET TO",
            nbFeatures, "\n")
    }
    # loop over grid of possible regularization values (lasso)
    # --> note: either nbFeatures.vect or normFraction.vect contains a single
    #           element
    stopifnot(length(normFraction.vect) == 1 || length(nbFeatures.vect) == 1)
    for (normFraction in normFraction.vect) {   
      if (verbose) {
          cat("      -- TUNING HYPERPARAMETER WITH normFraction SET TO",
              normFraction, "\n")
      }
      
      set.seed(42)
      # split current data subset (from outer CV loop) into inner CV sets
      cvSet.inner.list <- get.cvIndexSets(nrCVfolds = cvFoldInner,
                                          sampleNames = rownames(trainX),
                                          stratified = stratifiedCVsplit)

      # loop over grid of possible hyperparameter values
      for (param.inner.iter in 1:length(tuning.vals[[1]])) {
        param.inner <- tuning.vals[[1]][param.inner.iter]
        names(param.inner) <- names(tuning.vals)
        inner.res.counter <- inner.res.counter + 1
        # update inner CV runs summary table
        tuningRes.df[inner.res.counter,
                     c("nbFeatures", "normFraction", "parameter")] <-
                         c(nbFeatures, normFraction, param.inner)
        cvSet.inner.counter <- 0
        # loop over all inner CV sets
        for (cvSet.inner in cvSet.inner.list) {
          # keep track of which training set is being processed
          cvSet.inner.counter <- cvSet.inner.counter + 1
          if (verbose) {
              cat("PROCESSING INNER CV SET", cvSet.inner.counter, "OUT OF",
                  length(cvSet.inner.list), "\n")
          }
          # format data to use it with the CMA package
          trainY.inner <- trainY[cvSet.inner[["trainSampleIndic"]]]
          trainX.inner <- trainX[cvSet.inner[["trainSampleIndic"]], ]
          trainX.inner.scaled <- scaling(trainX.inner,
                                         scaling.method = scaling.method)$X.scaled
          # perform variable ranking
          withCallingHandlers(
            varsel.obj.train.inner <-
              GeneSelection(X = trainX.inner.scaled,
                            y = trainY.inner,
                            method = featureSelectionMethod,
                            norm.fraction = normFraction),
              warning = learningSetsNotProvidedWarning)
          # evaluate classifier on validation set (from inner CV sets!)
          perf.testset.inner <-
            validate.classifier(X.training = t(trainX),
                                Y.training = trainY,
                                training.ind = cvSet.inner[["trainSampleIndic"]],
                                classifier = classifier,
                                parameter.opt = param.inner,
                                features = toplist(varsel.obj.train.inner,
                                  k = min(c(nbFeatures,
                                      sum(varsel.obj.train.inner@importance[[1]] >
                                          0))), show = FALSE)[,"index"],
                                nb.features.on.array = nbFeaturesOnArray,
                                scaling.method = scaling.method)
          # update inner CV runs summary table
          tuningRes.df[inner.res.counter,
                       paste("errInner", cvSet.inner.counter, sep = "")] <-
                           perf.testset.inner["misclassification"]
          
        } # end loop over inner CV sets
      } # end loop over hyperparameter value grid
    } # end loop over norm fraction grid
  } # end loop over nb feature grid
                  
  # update inner CV runs summary data frame:
  # --> compute average performance over all (inner) CV test sets
  tuningRes.df[, "errAvg"] <- rowMeans(tuningRes.df[, -c(1:3)])  
        
  # keep best value:
  # --> best average performance on inner CV
  # --> if ex-aequo, keep parameter value leading to smaller model:
  #     1) small number of features / large norm fraction
  #     2) low complexity
  indic.min.err <- which(tuningRes.df[, "errAvg"] ==
                         min(tuningRes.df[, "errAvg"], na.rm = TRUE))
  indic.few.var <- which((tuningRes.df[indic.min.err, "nbFeatures"] -
                          tuningRes.df[indic.min.err, "normFraction"]) ==
                         min(tuningRes.df[indic.min.err, "nbFeatures"] -
                             tuningRes.df[indic.min.err, "normFraction"]))
  if (favorSmallModel == "min") {
      opt.line <- indic.min.err[indic.few.var][which.min(
          tuningRes.df[indic.min.err, "parameter"][indic.few.var])]
  } else {
      opt.line <- indic.min.err[indic.few.var][which.max(
          tuningRes.df[indic.min.err, "parameter"][indic.few.var])]
  }

  # select optimal hyperparameter value and nb of features from summary
  # data frame of inner CV loop
  nbFeatures.opt <- tuningRes.df[opt.line, "nbFeatures"]
  normFraction.opt <- tuningRes.df[opt.line, "normFraction"]
  parameter.opt <- tuningRes.df[opt.line, "parameter"]
  names(parameter.opt) <- names(tuning.vals)

  # now we use the outer CV sets to get a performance estimate for the
  # classifier with the selected optimal hyperparameter value and number
  # of parameters (or norm fraction)
  # => learn classifier of each outer CV training set and compute performance 
  #    on corresponding outer CV test set

  # --> scale training data
  trainX.scaled <-
    scaling(trainX,
            scaling.method = scaling.method)$X.scaled

  # --> order features
  withCallingHandlers(
    varsel.obj.train <-
      GeneSelection(X = trainX.scaled, y = trainY,
                    method = featureSelectionMethod,
                    norm.fraction = normFraction.opt),
      warning = learningSetsNotProvidedWarning)

  # --> train and evaluate classifier
  perf.testset <-
    validate.classifier(X.training = X.bc,
                        Y.training = sampleType,
                        training.ind = cvSet[["trainSampleIndic"]],
                        classifier = classifier,
                        features = toplist(varsel.obj.train,
                            k = min(c(nbFeatures.opt,
                                sum(varsel.obj.train@importance[[1]] >
                                    0))), show = FALSE)[,"index"],
                        parameter.opt = parameter.opt,
                        nb.features.on.array = nbFeaturesOnArray,
                        scaling.method = scaling.method)
  
  # update outer CV runs summary data frame
  cvSet.lab <- paste("cvSet", cvSetCounter, sep = "")
  cvValidation.df[cvSet.lab, "nbFeatures"] <- nbFeatures.opt
  cvValidation.df[cvSet.lab, "normFraction"] <- normFraction.opt
  cvValidation.df[cvSet.lab, "selectedFeatures"] <-
      paste(rownames(X.bc)[toplist(varsel.obj.train,
                                   k = min(c(nbFeatures.opt,
                                       sum(varsel.obj.train@importance[[1]] >
                                           0))), show = FALSE)[,"index"]],
            collapse = ";")
  cvValidation.df[ cvSet.lab, "optimalParameter"] <- parameter.opt
  stopifnot(all(names(perf.testset) %in% colnames(cvValidation.df)))
  cvValidation.df[cvSet.lab, names(perf.testset)] <- perf.testset
} # end loop over outer CV sets

# NOTES:
# --> the CV estimate of the classifier performance is obtained by averaging
#     the performance estimate obtained on the outer CV test sets
# --> the CV estimate is not a value for a specific classifier, since the
#     trained models can be different for each outer CV training set (different
#     set of selected variables)
# --> however, all trained models use the same hyperparameter value and the
#     same number of features
# --> so basically it estimates the performance that can be obtained by
#     constructing a classifier with the above nested CV procedure
```

### Learn classifier to apply to external validation data sets

In addition to the optimal hyperparameter value and the optimal number of features for our classifier, the code above provides a CV estimate of the classification error. However, it does not yet provide a classifier which can be applied on external validation data. To get such an object, we would like to work with as many samples as possible. Hence, we learn a classifier on the whole training data set.

The internal performance estimate computed above assesses the performance of a classifier learnt based on the inner CV scheme. Hence, we decided to proceed in the same way for the external validation:

- train a classifier based on an "inner" CV to select the optimal hyperparameter value and number of features
- apply this classifier to the external validation data set to get the "true" performance estimate

```
# summary data frame to store outcome for each CV set
tuningRes.df.final <-
  as.data.frame(matrix(NA, nrow = length(nbFeatures.vect) *
                       length(normFraction.vect) * length(tuning.vals[[1]]),
                       ncol = 3 + cvFoldOuter,
                       dimnames = list(NULL,
                           c("nbFeatures", "normFraction", "parameter",
                             paste(rep("errOuter", cvFoldOuter),
                                   1:cvFoldOuter, sep = "")))))

outer.res.counter <- 0
set.seed(42) 
# loop over grid of number of features (wilcoxon test)
for (nbFeatures in nbFeatures.vect) {   
  if (verbose) {
    cat("   - OUTER: TUNING HYPERPARAMETER WITH nbFeatures SET TO",
        nbFeatures, "\n")
  }
  # loop over grid of regularization values (lasso)
  for (normFraction in normFraction.vect) {   
    if (verbose) {
      cat("      -- OUTER: TUNING HYPERPARAMETER WITH normFraction SET TO",
          normFraction, "\n")
    }
    # loop over grid of hyperparameter values
    for (param.outer.iter in 1:length(tuning.vals[[1]])) {
      param.outer <- tuning.vals[[1]][param.outer.iter]
      names(param.outer) <- names(tuning.vals)
      outer.res.counter <- outer.res.counter + 1
      # update summary data frame
      tuningRes.df.final[outer.res.counter,
                         c("nbFeatures", "normFraction",
                           "parameter")] <-
                               c(nbFeatures, normFraction,
                                 param.outer)
      cvSet.outer.counter <- 0
      # loop over CV sets
      for (cvSet.outer in cvSet.list) {
        # keep track of which training set is being processed
        cvSet.outer.counter <- cvSet.outer.counter + 1
        if (verbose) {
            cat("OUTER: PROCESSING OUTER CV SET",
                cvSet.outer.counter,
                "OUT OF", length(cvSet.list), "\n")
        }                    
        # format data such as to be able to use it with CMA package functions
        trainY.outer <- sampleType[cvSet.outer[["trainSampleIndic"]]]
        trainX.outer <- t(X.bc)[cvSet.outer[["trainSampleIndic"]], ]
        # scale the training data
        trainX.outer.scaled <- scaling(trainX.outer,
                                       scaling.method = scaling.method)$X.scaled
        # order the features
        withCallingHandlers(
            varsel.obj.train.outer <-
            GeneSelection(X = trainX.outer.scaled, y = trainY.outer,
                          method = featureSelectionMethod,
                          norm.fraction = normFraction),
            warning = learningSetsNotProvidedWarning)
        # build (on train CV set) and evaluate (on test CV set) the classifier
        perf.testset.outer <-
            validate.classifier(X.training = X.bc,
                                Y.training = sampleType,
                                training.ind = cvSet.outer[["trainSampleIndic"]],
                                classifier = classifier,
                                features = toplist(varsel.obj.train.outer,
                                  k = min(c(nbFeatures,
                                    sum(varsel.obj.train.outer@importance[[1]] >
                                        0))), show = FALSE)[,"index"],
                                parameter.opt = param.outer,
                                nb.features.on.array = nbFeaturesOnArray,
                                scaling.method = scaling.method)
        
        # update summary data frame
        tuningRes.df.final[outer.res.counter,
                           paste("errOuter", cvSet.outer.counter, sep = "")] <-
                             perf.testset.outer["misclassification"]
      } # end cv loop
    } # end hyperparameter loop
  } # end normfraction loop
} # end nbfeature loop


# summary data frame:
# --> compute average performance over all CV test sets# 
tuningRes.df.final[, "errAvg"] <- rowMeans(tuningRes.df.final[, -c(1:3)])  
        
        
# keep best value:
# --> best average performance on inner CV
# --> if ex-aequo, keep parameter value leading to smaller model:
#     1) small number of features / large norm fraction
#     2) low complexity
indic.min.err <- which(tuningRes.df.final[, "errAvg"] ==
                       min(tuningRes.df.final[, "errAvg"], na.rm = TRUE))
indic.few.var <- which((tuningRes.df.final[indic.min.err, "nbFeatures"] -
                        tuningRes.df.final[indic.min.err, "normFraction"]) ==
                       min(tuningRes.df.final[indic.min.err, "nbFeatures"] -
                           tuningRes.df.final[indic.min.err, "normFraction"]))
if (favorSmallModel == "min") {
  opt.line <-
    indic.min.err[indic.few.var][which.min(
        tuningRes.df.final[indic.min.err,
                           "parameter"][indic.few.var])]
} else {
  opt.line <- indic.min.err[indic.few.var][which.max(
    tuningRes.df.final[indic.min.err,
                       "parameter"][indic.few.var])]
}
            
# select optimal hyperparameter value and nb of features from summary
# data frame of CV loop   
nbFeatures.opt.final <- tuningRes.df.final[opt.line, "nbFeatures"]
normFraction.opt.final <- tuningRes.df.final[opt.line, "normFraction"]
parameter.opt.final <- tuningRes.df.final[opt.line, "parameter"]
names(parameter.opt.final) <- names(tuning.vals)

# prepare objects to learn final classifier (with hyperparameter
# parameter.opt.final and number of features set according to
# nbFeatures.opt.final or normFraction.opt.final
# --> training data
tX.bc.scaled <- scaling(t(X.bc), scaling.method = scaling.method)$X.scaled
# --> order features
withCallingHandlers(
  varsel.obj.train <- GeneSelection(X = tX.bc.scaled, y = sampleType,
                                    method = featureSelectionMethod,
                                    norm.fraction = normFraction.opt.final),
  warning = learningSetsNotProvidedWarning)
# --> select features            
features.indic.final <- toplist(varsel.obj.train,
                                k = min(c(nbFeatures.opt.final,
                                    sum(varsel.obj.train@importance[[1]] >
                                        0))), show = FALSE)[,"index"]
```

## External validation of classifiers

```
# evaluate classifier on external validation data sets
# --> combine training and validation data together
X.inp.noEffect <- cbind(X.bc, X.val.noEffect)
X.inp.effect <- cbind(X.bc, X.val.effect)
Y.inp <- c(sampleType, sampleType.val)
            
# --> build (on training data) and evaluate (on balanced data without effect)
#     the classifier 
(cl.performance.b_ne.final <-
 validate.classifier(X.training = X.inp.noEffect, Y.training = Y.inp,
                     training.ind = 1:ncol(X.bc), classifier = classifier,
                     features = features.indic.final,
                     parameter.opt = parameter.opt.final,
                     nb.features.on.array = nbFeaturesOnArray,
                     scaling.method = scaling.method))

# --> build (on training data) and evaluate (on balanced data with effect)
#     the classifier 
(cl.performance.b_e.final <-
 validate.classifier(X.training = X.inp.effect, Y.training = Y.inp,
                     training.ind = 1:ncol(X.bc), classifier = classifier,
                     features = features.indic.final,
                     parameter.opt = parameter.opt.final,
                     nb.features.on.array = nbFeaturesOnArray,
                     scaling.method = scaling.method))
```

## Defined functions and (explicitly) loaded packages

### Loaded R packages

```
# load all required packages
require(plsgenomics)
require(glmnet)
require(lattice)
require(randomForest)
require(MASS)
require(class)
require(mgcv)
require(e1071)
require(Biobase)
require(corpcor)
require(BiocGenerics)
require(Matrix)
require(sva)                           
require(CMA)
```

### Function to simulate the data

```
##' This function simulates (log2-transformed) microarray gene expression
##' data based on a real data matrix.
##'
##' @title : Simulate mircroarray gene expression data . 
##' @param X : expression matrix
##' @param g1b1 : number of group1 samples in batch1 (training set)
##' @param g2b1 : number of group2 samples in batch1 (training set)
##' @param g1b2 : number of group1 samples in batch2 (training set)
##' @param g2b2 : number of group2 samples in batch2 (training set)
##' @param g1v : number of group1 samples in the validation set
##' @param g2v : number of group2 samples in the validation set
##' @param batch : strength of the batch effect: 'no', 'small', 'same' or
##' 'large'
##' @param min.effect : minimal effect size
##' @param replace : set to @code{TRUE} to sample from @code{X} with
##' replacement (default is @code{FALSE})
##' @param logarithmize : set to @code{TRUE} if \code{X} should be log2
##' transformed (default = @code{FALSE})
##' @param exp.rate : rate for the exponential distributions: used for
##' simulation of differential expression and batch effect (default =
##' @code{1.5})
##' @param de.indic : logical vector indicating which features 
##' will be differentially expressed in the simulated data matrix 
##' @param de.sign : vector defining the sign of the differential
##' expression for each feature
##' @param batch.indic : logical vector indicating which features will
##' be affected by batch effect in the simulated data matrix
##' @param batch.sign : vector defining the sign of the batch effect for
##' each feature
##' @return : A list with
##' \begin{itemize}
##'   \item @code{X.sim} :
##'   \item @code{X.val} :
##'   \item @code{de.effect} :
##'   \item @code{batch.effect} : 
##' \end{itemize}
##' @author : Charlotte Soneson and Sarah Gerster
sim.real <- function(X, g1b1, g2b1, g1b2, g2b2, g1v, g2v,
                     batch, min.effect, replace = FALSE,
                     logarithmize = FALSE, exp.rate = 1.5,
                     de.indic, de.sign, batch.indic, batch.sign) {
  # ensure that we have samples as columns, genes as rows
  if (nrow(X) < ncol(X)) X <- t(X)
  
  # sample columns from X
  X.all <- X[, sample(1:ncol(X), (g1b1 + g2b1 + g1b2 + g2b2 + g1v + g2v),
    replace)]
  
  # if 'logarithmize' == TRUE, log2-transform sampled values
  if (logarithmize) X.all <- log2(X.all)
  
  # split samples into categories (control/case and batch1/batch2)
  categ <- factor(rep(c('g1b1', 'g2b1', 'g1b2', 'g2b2', 'g1v', 'g2v'),
                     c(g1b1, g2b1, g1b2, g2b2, g1v, g2v)))
  split.ind <- split(1:ncol(X.all), categ)
  X.all.list <- lapply(split.ind, FUN = function(z) { X.all[, z] })
  
  # set the mean of each category to the same value
  overall.mean <- rowMeans(X.all)
  X.all.list.samemean <- lapply(X.all.list,
    FUN = function(z) { z - rowMeans(z) + overall.mean })

  # set the batch effect
  de.mu <- min.effect + rexp(nrow(X.all), rate = exp.rate)
  if (batch == 'no') {
    batch.mu <- rep(0, nrow(X.all))
  } else if (batch == 'small') {
    batch.mu <- 0.5 * min.effect + rexp(nrow(X.all), rate = exp.rate)
  } else if (batch == 'same') {
    batch.mu <- min.effect + rexp(nrow(X.all), rate = exp.rate)
  } else if (batch == 'large') {
    batch.mu <- 2 * min.effect + rexp(nrow(X.all), rate = exp.rate)
  } else {
    stop("unknown batch effect level")
  }
  
  # set the differential expression (control/case effect)
  de.effect.matrix.g2v <-
    matrix((1-rlnorm(nrow(X.all.list.samemean$g2v) *
                     ncol(X.all.list.samemean$g2v),
                     meanlog = -0.5, sdlog = 0.4)),
           nrow(X.all.list.samemean$g2v), ncol(X.all.list.samemean$g2v))
  if (!is.null(X.all.list.samemean$g2b1)){
    de.effect.matrix.g2b1 <-
      matrix((1-rlnorm(nrow(X.all.list.samemean$g2b1) *
                       ncol(X.all.list.samemean$g2b1),
                       meanlog = -0.5, sdlog = 0.4)),
             nrow(X.all.list.samemean$g2b1), ncol(X.all.list.samemean$g2b1))
  } else {
    de.effect.matrix.g2b1 <- numeric()
  }
  if (!is.null(X.all.list.samemean$g2b2)){
    de.effect.matrix.g2b2 <-
      matrix((1-rlnorm(nrow(X.all.list.samemean$g2b2) *
                       ncol(X.all.list.samemean$g2b2),
                       meanlog = -0.5, sdlog = 0.4)),
             nrow(X.all.list.samemean$g2b2), ncol(X.all.list.samemean$g2b2))
  } else {
    de.effect.matrix.g2b2 <- numeric()
  }

  # add batch effect and differential expression to data matrices
  X.all.list.effects <- NULL
  X.all.list.effects$g1b1 <- X.all.list.samemean$g1b1
  X.all.list.effects$g1v <- X.all.list.samemean$g1v
  X.all.list.effects$g2b1 <-
    X.all.list.samemean$g2b1 +
      de.effect.matrix.g2b1 * de.indic * de.sign * de.mu
  X.all.list.effects$g2v <-
    X.all.list.samemean$g2v +
      de.effect.matrix.g2v * de.indic * de.sign * de.mu
  X.all.list.effects$g1b2 <-
    X.all.list.samemean$g1b2 + batch.indic * batch.sign * batch.mu
  X.all.list.effects$g2b2 <-
    X.all.list.samemean$g2b2 +
      de.effect.matrix.g2b2 * de.indic * de.sign * de.mu +
        batch.indic * batch.sign * batch.mu
  
  # add noise 
  X.all.list.effects.noise <-
    lapply(X.all.list.effects,
           FUN = function(z) { if (length(z)!=0) z + matrix(rnorm(length(z),
                                           mean = 0, sd = 2),
                                           nrow(z), ncol(z)) })
  
  # put training data together in one matrix
  X.sim <- cbind(X.all.list.effects.noise$g1b1,
                 X.all.list.effects.noise$g2b1, 
                 X.all.list.effects.noise$g1b2,
                 X.all.list.effects.noise$g2b2)
  colnames(X.sim) <-
    paste(as.character(categ)[1:(g1b1 + g1b2 + g2b1 + g2b2)],
          c(if(g1b1 > 0) {1:g1b1}, if(g2b1 > 0) {1:g2b1},
            if(g1b2 > 0) {1:g1b2}, if(g2b2 > 0) {1:g2b2}), sep = '.')
  
  # combine validation data in a matrix
  X.val <- cbind(X.all.list.effects.noise$g1v,
                 X.all.list.effects.noise$g2v)
  colnames(X.val) <- 
    paste(as.character(categ)[(1 + g1b1 + g1b2 + g2b1 +
                               g2b2):(g1v + g2v + g1b1 + g1b2 +
                                      g2b1 + g2b2)],
          c(if(g1v > 0) {1:g1v}, if(g2v > 0) {1:g2v}), sep = '.')

  # return matrix with simulated intensities (test and validation sets),
  # indication of probesets which are affected by differential expression
  # and indication of probesets which are affected by batch effect   
  return(list(X.sim = X.sim, X.val = X.val,
              de.effect = de.indic * de.sign * de.mu,
              batch.effect = batch.indic * batch.sign * batch.mu))
}
```

### Altered definition of the knn function

```
##' add argument ... to be able to provide additional arguments (for
##' compatibility with calls to other classifiers)
##' 
##' @title : knn (see knn from package class for details)
##' @param train : training data 
##' @param test : test data
##' @param cl : true classifications of training data
##' @param k : hyperparameter (number of neighbors)
##' @param l : minimum vote for definite decision
##' @param prob : if true, function returns proportion of votes
##' @param use.all : handling of ties (equal distances); if this is true, use
##' ##' all tied values
##' @param ... : additional arguments to hand over to other functions
##' @return : classification
##' @author : Charlotte Soneson and Sarah Gerster
knn <- function(train, test, cl, k = 1, l = 0, prob = FALSE,
                use.all = TRUE, ...) {
  class::knn(train, test, cl, k = 1, l = 0, prob = prob, use.all = use.all)
}
```

### Function to validate a classifier

```
##' Validate a classifier either in the outer CV loop or on external
##' data: train classifier with provided features and corresponding
##' optimized parameter values on training data, then validate on test
##' set. Return a selection of different performance measures.
##' 
##' @title : validate.classifier
##' @param X.training : matrix with data used for training. In case of
##' internal validation (in a CV scheme) this is the complete data
##' matrix with training and test data. In this case, the argument
##' training.ind has to be set to specify which samples belong to the
##' training set. This matrix is of the form features x samples.
##' @param Y.training : class label for the data in @code{X.training}:
##' vector of length @code{ncol(X.training)}
##' @param training.ind : indices of samples in the training set. Only
##' set this argument when doing internal (in a CV loop) validation,
##' i.e. when the provided @code{X.training} contains both, training
##' and test samples. Default is @code{NULL} (for external validation).
##' @param n.samples : setting about the data set to indicate which
##' external data set (one compatible with the training set) should be
##' loaded. Default is @code{NULL}.
##' @param balance : setting about the data set to indicate which
##' external data set (one compatible with the training set) should be
##' loaded. Default is @code{NULL}.
##' @param diff.expression : setting about the data set to indicate which
##' external data set (one compatible with the training set) should be
##' loaded. Default is @code{NULL}.
##' @param replicate : setting about the data set to indicate which
##' external data set (one compatible with the training set) should be
##' loaded. Default is @code{NULL}.
##' @param data.path : path where the validation set can be found
##' @param classifier : the classifier that should be evaluated
##' @param features : selected features to use in the final classifier
##' @param parameter.opt : optimized parameter values to use for the
##' final classifier
##' @param nb.features.on.array : number of features to take into
##' consideration among all simulated features. This argument is only
##' needed for the external validation. Default value is @code{NULL}.
##' @param scaling.method : specify which, if any, scaling method should
##' be applied to the data before applying the classifier. Default is
##' NULL (no scaling).
##' @return : named list with sevral performance measures
##' @author : Charlotte Soneson and Sarah Gerster
validate.classifier <- function(X.training, Y.training, training.ind = NULL,
                                classifier, features, parameter.opt,
                                nb.features.on.array = NULL,
                                scaling.method = NULL) {
  # CV validation
  X <- X.training
  sample.type <- Y.training

  # scaling the expression matrix
  # --> memorize scaling parameters from training set
  tX.training.scaled.list <-
    scaling(t(X[, training.ind]), scaling.method = scaling.method)
  # --> apply scaling parameters from training set to test set
  tX.test.scaled <-
    scaling(t(X[, -training.ind]), scaling.method = scaling.method,
            param.list = tX.training.scaled.list$param.list)$X.scaled
  # --> combine scaled values in a single matrix
  tX.scaled <- matrix(NA, ncol = nrow(X), nrow = ncol(X))
  tX.scaled[training.ind, ] <- tX.training.scaled.list$X.scaled
  tX.scaled[-training.ind, ] <- tX.test.scaled
  
  # classify
  # --> depending on value of 'classifier', apply selected classifier
  if( classifier == "svmCMA") {    
    cl.res <-
      eval(parse(text = paste(classifier,
                   "(X = tX.scaled[, features, drop = FALSE],",
                   "y = sample.type,",
                   "cost = ", parameter.opt, ", ",
                   "learnind = training.ind,",
                   "probability = FALSE, seed = 42, models = TRUE)",
                   sep = "")))
  } else if (classifier == "rfCMA") {
    if (length(features) > 1) {
      cl.res <-
        eval(parse(text = classifier))(
                     X = tX.scaled[, features, drop = FALSE],
                     y = sample.type,
                     learnind = training.ind,
                     seed = 42, models = TRUE,
                     ntrees = parameter.opt)
    } else {
      cl.res <- NULL
    }
  } else if (classifier == "plrCMA") {
    cl.res <-
      eval(parse(text = classifier))(
                   X = tX.scaled[, features, drop = FALSE],
                   y = sample.type,
                   learnind = training.ind,
                   models = TRUE,
                   probability = TRUE,
                   lambda = parameter.opt)     
  } else if (classifier == "knnCMA") {
    cl.res <-
      eval(parse(text = classifier))(
                   X = tX.scaled[, features, drop = FALSE],
                   y = sample.type,
                   learnind = training.ind,
                   models = TRUE,
                   k = parameter.opt)     
  } else {
    stop("Classifier not supported.")
  }
    
  # evaluation
  if (!is.null(cl.res)) {
    # --> provide a set of chosen performance measures (if available for
    #     the selected classifier)
    performance <- c(specificity = evaluation(list(cl.res), scheme = "iter",
                       measure = "specificity")@score,
                     sensitivity = evaluation(list(cl.res),
                       scheme = "iter", measure = "sensitivity")@score,
                     misclassification = evaluation(list(cl.res),
                       scheme = "iter", measure = "misclassification")@score,
                     average.prob = ifelse(classifier %in% c("svmCMA", "knnCMA"),
                       NA, evaluation(list(cl.res), scheme = "iter",
                                      measure = "average probability")@score),
                     auc = ifelse(classifier %in% c("svmCMA", "knnCMA"), NA,
                       evaluation(list(cl.res), scheme = "iter",
                                  measure = "auc")@score))
  } else {
    # --> if no classifier could be learnt for this specific choice of
    #     parameters
    performance <- c(specificity = NA, sensitivity = NA,
                     misclassification = NA, average.prob = NA,
                     auc = NA)
  }
  return(performance)
}
```

### Function to scale the data

```
##' Function to apply different scaling schemes to a matrix X.
##'
##' @title : scaling
##' @param X : matrix to be scaled: samples x features
##' @param scaling.method : saling method to use. Can be NULL (no scaling,
##'   zscore or range01. Default is NULL.
##' @param param.list : named list with parameter values to use for the
##'   specified scaling method. Default is NULL: parameters are then learnt
##'   on the provided data
##' @return : named list with scaled matrix, saling method and parameters
##'   used for the scaling
##' @author : Charlotte Soneson and Sarah Gerster
scaling <- function(X, scaling.method = NULL, param.list = NULL) {
  # make sure the matrix is in the form sample x features
  stopifnot(ncol(X) > nrow(X))
  if (is.null(scaling.method)) {
    # no scaling
    X.scaled <- X
  } else  if (scaling.method == "zscore") {
    # apply z-transform: substract mean and divide by standard deviation
    if (is.null(param.list)) {
      # learn mean and sd on current data
      X.scaled <- scale(X, center = TRUE, scale = TRUE)
      param.list <- list(m = attr(X.scaled, "scaled:center"),
                         s = attr(X.scaled, "scaled:scale"))
    } else {
      # apply provided parameters
      X.scaled <- scale(X, center = param.list$m, scale = param.list$s)
    }
  } else if (scaling.method == "range01") {
      # scale data to have transformed data in the intervall [0, 1]
    if (is.null(param.list)) {
      # learn parameters on current data
      col.max <- apply(X, 2, max, na.rm = TRUE)
      col.min <- apply(X, 2, min, na.rm = TRUE)
      scale.factor <- 1.0 / (col.max - col.min)
      X.scaled <- X
      for (i in 1:dim(X.scaled)[2]) {
        X.scaled[, i] <-
          (X.scaled[, i] - col.min[i]) * scale.factor[i]
      }
      param.list <- list(col.min = col.min, col.max = col.max)
    } else {
      # apply provided parameters
      col.min <- param.list$col.min
      col.max <- param.list$col.max
      scale.factor <- 1.0 / (col.max - col.min)
      X.scaled <- X
      for (i in 1:dim(X.scaled)[2]) {
        X.scaled[, i] <-
          (X.scaled[, i] - col.min[i]) * scale.factor[i]
      }
    }
  } else {
    stop("Scaling method not implemented.")
  }

  # return list with scaled matrix, applied parameters and applied
  # scaling method
  return(list(X.scaled = X.scaled, param.list = param.list,
              scaling.method = scaling.method))
}
```

### Function to generate sample indices of CV sets

```
##' Generate a list with indices of the test samples for each CV fold.
##' 
##' @title : get.cvTestSamp.list
##' @param nrCVfolds : number of CV sets 
##' @param sampleNames : names of the samples, allowing to split them into
##'   g1 and g2 (control and case)
##' @param stratified : set to FALSE to disable stratified sampling to build
##'   the sets of test samples (default is TRUE)
##' @param setName : base name to use when naming the CV sets (items of the
##'   returned list)
##' @return : named list with test indices for each CV set
##' @author : Charlotte Soneson and Sarah Gerster
get.cvTestSamp.list <- function(nrCVfolds, sampleNames,
                                stratified = TRUE, setName = "cvSet") { 
  # separate samples according to their type: control/case <-> g1/g2
  samp.g1 <- sampleNames[which(sapply(sampleNames,
                                      function(x) substr(x, 1, 2)) == "g1")]
  samp.g2 <- sampleNames[which(sapply(sampleNames,
                                      function(x) substr(x, 1, 2)) == "g2")]

  # draw from samples: first all controls, then all cases
  if (stratified) {
    samp.sampled <- c(sample(samp.g1, size = length(samp.g1), replace = FALSE),
                      sample(samp.g2, size = length(samp.g2), replace = FALSE))
  } else {
    samp.sampled <- sample(c(samp.g1, samp.g2), replace = FALSE)
  }

  # go through the drawn samples, and distribute them one by one in the
  # different CV sets
  cvSet.samp.list <-
    lapply(1:nrCVfolds,
           function(i, ref.samp) ref.samp[i - 1 +
                                          seq(1, length(sampleNames) + 1 -
                                              nrCVfolds,
                                              by = nrCVfolds)],
           ref.samp = samp.sampled)

  # name the CV sets
  names(cvSet.samp.list) <- paste(setName, 1:nrCVfolds, sep = "")

  return(cvSet.samp.list)
}
```

### Function to generate a list of CV sets

```
##' Prepare list with CV sets: each list item is a list itself containing
##' the training and the test indices, respectively.
##' 
##' @title : get.cvIndexSets 
##' @param nrCVfolds : number of main CV sets
##' @param sampleNames : names of the samples, used to sample stratified CV sets
##' @param stratified : set to @code{FALSE} if you do not want to draw the CV
##'   sets in a stratified manner (default is TRUE)
##' @return : nested list of CV sets. Each list element provides the indices of
##'   the corresponding training and test set.
##' @author : Charlotte Soneson and Sarah Gerster
get.cvIndexSets <- function(nrCVfolds, sampleNames, stratified = TRUE) {
  cvTestSample.list <- get.cvTestSamp.list(nrCVfolds = nrCVfolds,
                                           sampleNames = sampleNames,
                                           stratified = stratified)
  # prepare list with CV sets: each list item is a list itself containing
  # the training and the test indices, respectively
  cvSets.list <- list()
  for (cvSet in names(cvTestSample.list)) {
    cvSets.list[[cvSet]] <- list()
    cvSets.list[[cvSet]][["testSampleIndic"]] <-
      which(sampleNames %in% cvTestSample.list[[cvSet]])
    cvSets.list[[cvSet]][["trainSampleIndic"]] <-
      which(!(sampleNames %in% cvTestSample.list[[cvSet]]))
  }

  return(cvSets.list)
}
```

### Function to suppress some warnings

```
##' Avoid displaying some warnings. They are unnecessary in our case, since
##' we do on purpose just what the functions warns us about.
##' 
##' @title : geneSelectionWarning
##' @param warn :warning message to potentially muffle 
##' @return : --
##' @author : Charlotte Soneson and Sarah Gerster
learningSetsNotProvidedWarning <- function(warn) {
  if (any(grepl("Argument 'learningsets' is missing", warn))) {
    invokeRestart("muffleWarning")
  }
}
```

## R session information used to generate this document

```
## R version 3.0.1 (2013-05-16)
## Platform: x86_64-pc-linux-gnu (64-bit)
## 
## attached base packages:
## [1] stats     graphics  grDevices utils     datasets  base     
## 
## other attached packages:
## [1] knitr_1.5      Defaults_1.1-1
## 
## loaded via a namespace (and not attached):
## [1] evaluate_0.5.1 formatR_0.10   stringr_0.6.2  tools_3.0.1
```

## Makefile used for compilation of this document

HTMLS= documented\_code.html

all: $(HTMLS)

clean:

```
rm -f *.md
```

%.html:%.Rmd

```
Rscript -e "library(knitr); knit('$*.Rmd')" 

pandoc --toc -s $*.md -o $*.html
```

## pandoc version used for conversion to html of this document

pandoc 1.9.4.2

Compiled with citeproc-hs 0.3.4, texmath 0.6.0.6, highlighting-kate 0.5.1.
